# Electropolymerized Molecularly Imprinted Polymers Supported on Carbon-Based Materials for (Bio)sensing: Direct and Indirect Detection Strategies

**DOI:** 10.3390/bios16060350

**Published:** 2026-06-22

**Authors:** Sergio Espinoza-Torres, Astrid Choquehuanca-Azaña, Nathalia Florencia B. Azeredo, Marcos Rufino, Lucio Angnes

**Affiliations:** 1 Department of Fundamental Chemistry, Institute of Chemistry, University of São Paulo, Av. Prof. Lineu Prestes, 748, São Paulo 05508-000, SP, Brazil; 2 Faculty of Pharmaceutical Sciences, Faculdades Oswaldo Cruz, Rua Brigadeiro Galvão, 540, São Paulo 01151-000, SP, Brazil

**Keywords:** molecularly imprinted polymer, electropolymerization, carbon-based materials, electrochemical sensor

## Abstract

Molecularly imprinted polymers (MIPs) offer robust, cost-effective, and highly selective alternatives to fragile biological receptors. Specifically, electropolymerization has emerged as a versatile strategy that enables the precise, in situ formation of uniform MIP films directly on electrode surfaces. This review provides a comprehensive overview of electropolymerized MIPs (eMIPs) supported on advanced carbon-based materials for electrochemical (bio)sensing. We emphasize how the synergistic integration of eMIPs with carbonaceous architectures significantly enhances electron transfer, active surface area, and overall analytical sensitivity. Key fabrication aspects are systematically discussed, including monomer selection, electropolymerization parameters, and efficient template removal. A central aspect of this work is the critical categorization of sensing mechanisms into direct and indirect detection strategies. This distinction elucidates how eMIPs can quantify a broad spectrum of electroactive and non-electroactive targets in complex matrices, while strategically avoiding excessively high applied potentials. Finally, alongside outlining the transition of these systems into portable technologies, we address a critical shortcoming in the current literature: the urgent need for analytical standardization through the rigorous reporting of Imprinting and Selectivity Factors using Non-Imprinted Polymer (NIP) controls.

## 1. Introduction

The growing demand for sensitive, selective, and stable analytical devices has driven significant advancements in the design of novel sensing platforms [1]. Central to their performance is the recognition element, which must selectively interact with the analyte of interest while minimizing interference from complex sample matrices [2]. In this context, molecularly imprinted polymers (MIPs) have emerged as a promising class of synthetic receptors. MIPs have become essential tools across diverse fields, from medical diagnostics, environmental monitoring, food safety, and pharmaceutical analysis, due to their ability to provide rapid and accurate detection of target molecules [3,4].

MIPs are produced through a polymerization process in the presence of a target molecule [5]. After polymer formation, the template is removed, leaving behind tailor-made specific cavities that are complementary in size, shape, and functional groups to the target analyte [6,7]. This approach mimics the lock-and-key mechanism observed in natural biological systems, such as enzyme-substrate or antibody–antigen interactions. However, MIPs offer superior chemical and thermal stability, robustness, long shelf-life, and cost effective production [8]. These properties make MIPs particularly attractive for integration into electrochemical biosensors, enabling the specific detection of a wide range of target molecules, including peptides, proteins, and even whole cells.

Among the different methods of MIP synthesis, electropolymerization has gained increasing attention, especially for sensor applications, due to its ability to precisely control the thickness, morphology, and uniformity of the polymer film directly on the electrode surface [9]. This technique allows for in situ generation of the MIP layer under mild conditions, often using aqueous solvents and low monomer concentrations [10]. Moreover, electropolymerization enables fine-tuning of the sensor’s analytical properties by adjusting key electrochemical parameters, such as the applied potential, scan rate, and number of polymerization cycles [11].

To further enhance the sensitivity and signal transduction of electropolymerized MIP-based sensors, researchers have increasingly incorporated carbon-based nanomaterials as supporting substrates or composites [12]. Materials such as graphene, graphene oxide (GO), reduced graphene oxide (rGO), carbon nanotubes (CNTs), carbon black, and carbon quantum dots (CQDs) offer unique physicochemical properties, including high electrical conductivity, large specific surface area, chemical stability, and the ability to facilitate fast electron transfer [13]. These features are crucial for amplifying electrochemical signals and lowering the limit of detection (LOD), enabling the development of high-performance biosensors [14].

The strategic advantage of these carbonaceous materials becomes particularly evident when compared to other conventional supporting substrates used in eMIP sensors, such as noble metals, metal oxides, and pure conducting polymers. While noble metal electrodes (e.g., gold, platinum) offer excellent conductivity, their high cost, lower specific surface areas, and susceptibility to surface oxidation at anodic potentials restrict their broader application. Metal oxides often present lower intrinsic electrical conductivity without extensive doping and can be chemically unstable under the extreme pH conditions sometimes required for sensor operation or template removal. Similarly, while pure conducting polymers provide tunable electroactivity, they frequently suffer from poor mechanical stability and structural degradation, such as swelling or shrinking, during continuous electrochemical cycling. In contrast, advanced carbon materials uniquely combine an exceptionally wide potential window, superior mechanical robustness, and highly tunable surface chemistry. This versatility allows for facile functionalization, providing robust anchoring points for the eMIP layer and ensuring long-term stability superior to that of purely polymeric or metallic supports [5,15].

The synergy between electropolymerized MIPs (eMIPs) and carbon-based materials results in hybrid architectures that combine the molecular selectivity of the polymeric layer with the enhanced transduction and mechanical strength of carbon nanostructures [16]. Recent studies have demonstrated the successful application of these hybrid systems in detecting a wide range of analytes, including hormones, pharmaceuticals, pesticides, heavy metals, and pathogenic biomarkers [17]. Furthermore, advances in nanofabrication and surface functionalization techniques have enabled the rational design of hierarchical and multifunctional sensing platforms with improved performance [18].

Although several reviews have addressed molecularly imprinted polymers, electrochemical sensors, or carbon nanomaterials independently, a comprehensive discussion specifically focused on electropolymerized MIPs (eMIPs) supported on carbon-based materials remains limited. To fill this gap, this review focuses exclusively on the rational design, fabrication, and application of these synergistic platforms. First, we detail the intrinsic advantages of the electropolymerization process, covering monomer selection, template removal methods, and the step-by-step characterization of the eMIP and NIP interfaces. Subsequently, we systematically explore the incorporation of diverse multidimensional carbon substrates as foundational supports that enhance analytical performance. A core contribution of this work is the critical categorization of (bio)sensing applications into direct and indirect detection strategies. We clarify the mechanisms of direct detection for various electroactive targets, including drugs, environmental pollutants, and biological compounds. Furthermore, we extensively discuss indirect detection—utilizing soluble probes, anchored probes, or probe-less methods. Importantly, we highlight that indirect strategies are vital not only for non-electroactive biomolecules and proteins, but also serve as a strategic alternative for electroactive analytes to avoid the application of excessively high oxidation potentials. Ultimately, this review provides practical guidelines for selecting ideal electroanalytical techniques and concludes with a critical evaluation of current literature, emphasizing the necessity of reporting the Imprinting Factor (IF) and Selectivity Factor.

To ensure a transparent and comprehensive literature survey, the studies included in this review were systematically collected from major scientific databases, including ScienceDirect, Web of Science, Scopus, PubMed, SpringerLink, Wiley Online Library, ACS Publications, and MDPI. The literature search focused predominantly on articles published within the last five years, utilizing combinations of keywords such as “electropolymerized molecularly imprinted polymers”, “electrochemical MIP sensors”, “carbon nanotubes”, “graphene”, “carbon quantum dots”, “biochar”, “laser-induced graphene”, and “electrochemical biosensors”. Preference was given to peer-reviewed studies that report detailed sensor fabrication strategies, comprehensive electrochemical characterization, robust analytical performance metrics, and validation in real-sample applications.

## 2. Molecularly Imprinted Polymers (MIPs)

**Figure 1:**
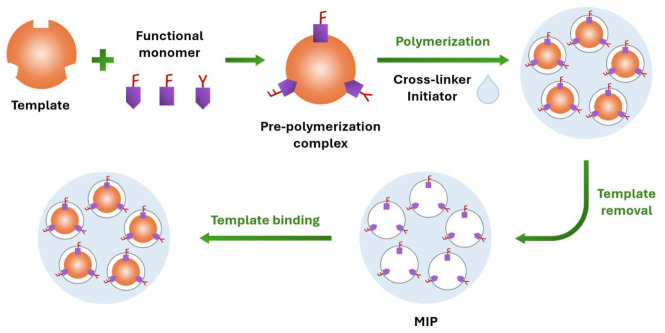
Fundamental steps involved in the synthesis of molecularly imprinted polymers (MIPs).

Molecularly imprinted polymers (MIPs) are defined by the International Union of Pure and Applied Chemistry (IUPAC) as synthetic polymers containing well-defined cavities (imprints) formed by template molecules, which mimic specific ligand–receptor interactions [19]. MIPs have emerged as attractive alternatives to biological recognition elements in sensor development. Their versatility allows for the detection of a broad spectrum of analytes, including small molecules, biomolecules, and pathogens. [20]. All synthesis methods follow a similar basic scheme: (1) polymer formation in the presence of a template molecule, covalently or noncovalently bound to functional groups; (2) template removal, generating complementary binding cavities; and (3) rebinding, in which the MIP selectively recognizes the target in complex samples (Figure 1) [21]. Various synthesis methods have been reported for MIPs, differing in reagent composition, polymerization conditions, and template removal strategies used to generate accessible binding cavities.

Free radical polymerization (FRP) is the most widely used method and is carried out under mild conditions, where it requires the presence of heat (bulk, precipitation, suspension) or UV-Vis radiation (photopolymerization) to initiate the reaction. The process involves mixing the template, functional monomer, cross-linker, initiator, and a porogen agent (a solvent or mixture of solvents) in a single vessel to produce a crosslinked polymer network. Advantages of this polymerization technique include simple, low-cost, minimal equipment requirements, and compatibility with a wide variety of functional monomers. However, it suffers from several significant shortcomings, such as: (i) irregular particle sizes and shapes caused by grinding; (ii) the partial destruction of binding sites during milling; (iii) labor-intensive and time-consuming post-polymerization processing; (iv) the need for large amounts of solvents for template removal; (v) low batch-to-batch reproducibility; and (vi) poor control over particle uniformity and surface area [11].

Others conventional methods offer specific advantages but also present limitations. Controlled radical polymerization (CRP) enables the precise regulation of polymer characteristics, such as molecular weight, distribution, and chain-end functionality, by utilizing activation-deactivation cycles that strictly control chain growth to produce well-defined macromolecular structures. However, CRP requires a strictly inert atmosphere due to its high sensitivity to oxygen. Furthermore, the use of transition metals as catalysts in the polymerization process can often be problematic due to the complexity and cost of metal extraction [22]. Alternatively, sol–gel polymerization is carried out at ambient temperature using water or ethanol as the reaction medium, avoiding toxic solvents and preventing chemical and thermal decomposition. Nevertheless, this method presents disadvantages in terms of its instability at basic pH, a more restricted list of functional monomer and sometimes poor template solubility on the used solvent [23].

In contrast, the electrochemical polymerization of monomers overcomes many of these limitations through the formation of conducting or non-conducting films directly on the electrode surface. This technique allows for precise control over film thickness through the adjustment of electrochemical parameters. Such control not only ensures the uniform deposition of the polymer onto carriers of various shapes and sizes but also reduces template leakage. Furthermore, the applied potential can influence monomer organization during polymer growth, enhancing the specificity of the resulting MIP and generating highly selective binding sites after template removal [10,11].

As shown in Table 1, electropolymerization simplifies the synthesis process by using fewer components than other methods. For example, it does not require chemical initiators because the electric potential directly generates reactive radicals in situ. Furthermore, while chemical methods require crosslinking agents, many electroactive monomers self-crosslink. Therefore, adding an external crosslinker is optional, although occasionally used to further modulate the structural rigidity of the MIP film, aiming for a more stable imprinted network with enhanced template recognition ability [24].

In summary, after evaluating various synthesis techniques, electropolymerization stands out as a highly advantageous approach for electrochemical sensor development. Its ability to generate films in situ allows precise control over film thickness while enhancing sensitivity and reproducibility. Therefore, its operational simplicity and capacity for material customization make electropolymerization a suitable strategy for fabricating high-performance sensing devices [20].

### 2.1. MIPs by Electropolymerization

Having established the distinct advantages of electropolymerized MIPs—such as the precise control over film thickness, high reproducibility, and the ability to grow the polymer network directly onto the transducer surface—it is essential to examine the systematic workflow required for their successful fabrication. The development of a highly selective electrochemical MIP sensor is a rational multistep process. It encompasses the fundamental understanding of reagent interactions, the precise control of the electrochemical synthesis, and the subsequent activation of the recognition cavities. Therefore, the following sections provide a structural analysis of these three critical stages: the computational and rational design of the pre-polymerization complex, the mechanisms of electrochemical polymerization, and the strategies for effective template removal.

### 2.2. Functional Monomer Selection: Chemical Interactions and Computational Modeling

**Figure 2:**
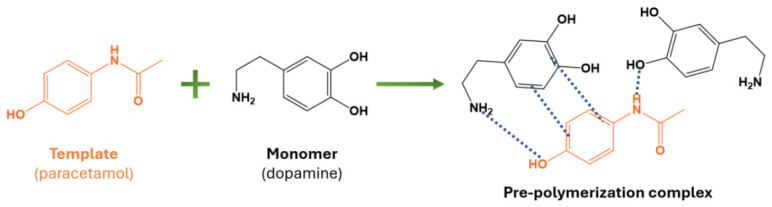
Key monomer-template interactions in the pre-polymerization complex.

As seen in Table 1, the electropolymerization process fundamentally involves two key components: template and functional monomer. The efficacy of molecular imprinting critically depends on forming a stable pre-polymerization complex (Figure 2) [23]. This approach, favored for its rapid binding and release kinetics, relies on multiple interactions (hydrogen bonds, ionic interactions, and π-π stacking) [25], which depends on the specific functional groups of the monomer (e.g., aniline, dopamine, *o*PD, phenol, EDOT) and the template.

To achieve a deeper mechanistic understanding of these interactions, Density Functional Theory (DFT) is increasingly utilized to model the pre-polymerization complex. DFT quantitatively estimates the ground-state interaction strength between the functional monomer (FM) and the template using the following equation:

(1) $$\Delta E=\left(E_{template}+E_{FM}\right)-E_{complex}$$

By calculating this thermodynamic stabilization (ΔE), where a more negative energy value denotes a stronger and more favorable interaction, computational models evaluate structural complementarity and accurately predict the optimal template-to-monomer ratio [26].

Complementing thermodynamic studies, frontier molecular orbital (FMO) analysis predicts molecular reactivity and physicochemical stability within the pre-polymerization complex [27]. The FMO analysis method is effective for evaluating intra and intermolecular interactions. The electron density delocalization between occupied and unoccupied orbitals indicates stable donor-acceptor interactions. A higher HOMO energy reflects a strong electron-donating capacity, while a lower LUMO energy indicates greater electron-accepting ability [28]. Furthermore, a narrowed HOMO-LUMO energy gap ($\Delta E_{GAP}$) upon complexation serves as a primary indicator of electronic softness and increased propensity for favorable template-monomer interactions [29].

The predictive power of these computational tools is well-documented. For instance, Karthika et al., validated the selection of pyrrole for a MIP sensor for Bisphenol A (BPA). They demonstrated the strong electrophilic nature of pyrrole, facilitating donor-acceptor interactions with the phenolic groups of BPA. Ultimately, the smaller $\Delta E_{GAP}$ for the BPA-pyrrole complex compared with the isolated components indicates its high stabilization via these favorable interactions [30]. Similarly, Rajaee et al. used spatial FMO mapping to evaluate pyrrole and acrylamide for the simultaneous recognition of biomarkers such as urea, creatinine, uric acid, and xanthine. They observed that LUMO of pyrrole is localized on its N–H group (enabling hydrogen bonding), while its HOMO covers the aromatic ring (facilitating π-π interactions). This dual-interaction profile perfectly complemented the frontier orbitals of the target templates. In contrast, the electron clouds of acrylamide lacked this distinct spatial separation. Consequently, precise FMO mapping justified selecting pyrrole over acrylamide to fabricate highly specific imprinted cavities [31].

Therefore, the proper selection of the functional monomer must be based on its capacity to provide chemical functionalities that are complementary to those of the target analyte, ultimately maximizing the association constant of the complex. This selection must carefully consider the chemical structure and properties of the template to ensure optimal interaction strength. Excessively weak interactions can lead to an insufficient number of well-defined imprinted cavities, thereby compromising specificity. Conversely, overly strong interactions can hinder the extraction of the template and its analogs from the polymer matrix, complicating both the template removal process and the regeneration of the recognition sites [32].

Beyond molecular interactions, incorporating solvent effects into DFT calculations via the Polarizable Continuum Model (PCM) is essential for a realistic representation of the electropolymerization environment. The choice of porogen is fundamental to the imprinting process since it dissolves polymerization agents and dictates the porous structure of the polymer matrix. In non-covalent imprinting, solvent polarity directly impacts the template-monomer interactions. Highly polar solvents actively compete for binding sites, often decreasing interaction energies (ΔE) compared to vacuum conditions [33,34]. Conversely, non-polar environments leave forces like hydrogen bonding largely undisturbed. Although non-polar solvents maximize binding affinity, polar solvents are frequently required for solubility [34]. Simulating varying dielectric constants is thus critical to prevent the overestimation of binding strengths, ensuring reliable predictions.

Salajegheh et al. used computational simulations to design a theophylline sensor, evaluating arginine, lysine, and phenylalanine as functional monomers. DFT binding energies (Equation (1)) identified arginine as the optimal monomer and predicted an ideal 1:4 template-to-monomer ratio. Furthermore, the gas phase optimized structures were subjected to PCM calculations to evaluate their respective solvation free energies ($\Delta G_{SOLV}$) in water. The resulting Gibbs free energy (ΔG) confirmed that the theophylline-(ARG)_4_ complex formation remained thermodynamically favorable in aqueous media. The fabricated sensor exhibited excellent analytical performance with a LOD an order of magnitude lower than previously reported devices, proving the efficacy of computational optimization [35].

In summary, the strong analytical performance of sensors designed using these tools demonstrates that computational screening effectively replaces the traditional trial-and-error approach, which is both time-consuming and reagent-intensive. Once the optimal system is theoretically established, the next crucial step is its physical synthesis, an area where electropolymerization emerges as a highly efficient technique that allows precise control over film formation.

### 2.3. Electrochemical Polymerization of Functional Monomers

**Figure 3:**
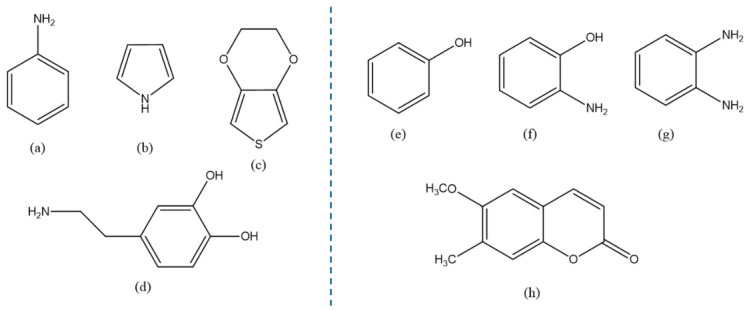
Monomers that polymerize into conductive films: (**a**) aniline, (**b**) pyrrole, (**c**) 3,4-ethylenedioxythiophene and (**d**) dopamine; and monomers that polymerize into non-conductive films: (**e**) phenol, (**f**) o-aminophenol, (**g**) o-phenylenediamine, and (**h**) scopoletin.

The electrochemical polymerization of monomers onto conducting substrates leads to the formation of either conducting or non-conducting polymer films (Figure 3), depending on the choice of monomer and the applied electropolymerization conditions. Monomers that yield electrically conducting polymers, such as aniline [36,37], pyrrole [38], 3,4-ethylenedioxythiophene (EDOT) [39], and dopamine [40], can be grown in thicker films, which is beneficial if the goal is to prepare complex 3D micro or nanostructured MIPs. In contrast, monomers that form insulating polymer films, such as phenol [41], *o*-aminophenol [42], *o*-phenylenediamine (oPD) [43], and scopoletin [44], exhibit a different behavior. As the insulating film grows, it progressively blocks the transfer of electrons between the electrode and the monomer in solution. Consequently, the film becomes sufficiently compact to hinder monomer permeation and suppress charge transfer, thereby terminating further polymer growth [25].

Because electrochemical oxidation occurs directly on the electrode surface, it leads to the formation of a film with an electrocontrollable thickness. The properties of the in situ fabricated film can be finely tuned by adjusting parameters during electrodeposition, such as the applied voltage, potential sweep rate, cycle duration, and material concentration. In particular, depositing thin MIP layers directly on the electrode surface displays great suitability for fabricating sensors with higher selectivity performance. Cyclic voltammetry (CV) is the predominant electrochemical technique used to generate these eMIPs. This technique allows precise control over polymer thickness, a key factor in the formation of recognition cavities, through parameters such as the number of cycles and an adequate potential window. Li et al. demonstrated this by developing an eMIP sensor for GHRP-6 via the electropolymerization of phenol. Although the electropolymerization of this non-conducting monomer intrinsically exhibits a self-limiting growth mechanism, precise cycle control is still required. By evaluating polymerization cycles ranging from 15 to 35, they observed that films formed with more than 20 cycles became very thick, hindering template extraction [41].

In contrast, conducting polymers, such as polypyrrole (PPy), tend to undergo continuous propagation. Because the growing conjugated backbone facilitates electron transfer, the polymer thickness typically increases proportionally with the number of voltammetric cycles or applied charge. This capacity for continuous growth is highly advantageous for creating thicker matrices capable of entrapping larger macromolecules. However, the electrical properties of the conductive polymer depend on the nature of the doping. A suitable doping agent improves conductivity and significantly increases polymer growth, whereas a dopant providing low conductivity impedes it [45]. Alternatively, this growth can be controlled electrochemically. For instance, potentiostatic electropolymerization was employed by Turco et al. By applying a constant potential of 0.75 V vs. SCE for 600 s, they deposited pyrrole in its overoxidized form. This electrochemically self-limits the polymer thickness, ensuring the formation of an optimally thin and highly permeable imprinting layer [46]. Moreover, beyond morphological control, pyrrole-based matrices offer excellent chemical recognition; besides π-π interactions, the amine group present on the polypyrrole ring facilitates electrostatic interactions, which significantly enhances the selectivity of the MIP and reduces its sensitivity to potential interferents [10].

Ultimately, successful electrodeposition yields a tailored matrix that securely encapsulates the target analyte. However, the sensor remains inactive since these recognition sites are fully occupied. Transforming this composite into a functional device requires thoroughly extracting the template. This next critical phase—template removal—demands a delicate balance: extraction strategies must be robust enough to disrupt the established non-covalent interactions while remaining sufficiently mild to preserve the structural integrity of the formed polymeric network.

### 2.4. Template Removal

To ensure optimal sensitivity and specificity, the template must be effectively removed. This step is essential to expose the selective binding cavities that are complementary in shape, size, and functional groups to the target molecule, enabling the MIP to perform effective molecular recognition. The primary objectives of the template removal process are (i) to completely clear the template molecule without damaging the polymer or binding sites; (ii) to prevent template bleeding (leaching during use); and (iii) to preserve the structural fidelity of the imprinted cavities for high selectivity and sensitivity [17]. Extraction strategies include the use of organic, acidic, or basic solvents, as well as electrochemical techniques like overoxidation.

**Figure 4:**
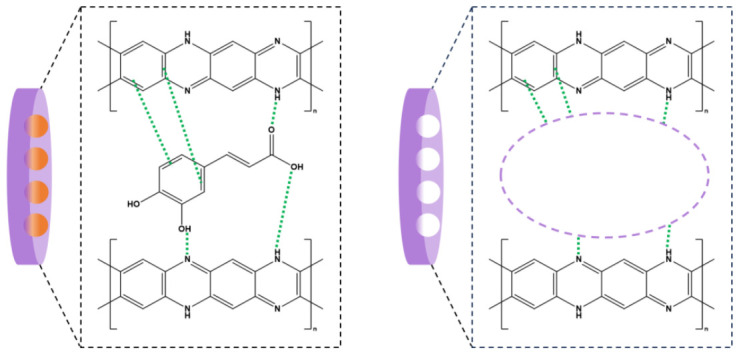
Template removal by extraction with solvents.

Solvent extraction: As the most widely used method, this approach involves immersing the MIP in a solvent system. It exploits the selective solubility of the template in a specific solvent or mixture, while keeping the polymer network insoluble and stable (Figure 4). A mixture of methanol and acetic acid is extensively employed for template extraction due to its high extraction efficiency and its capacity to solubilize most molecules, including unreacted residues [47]. Alternatively, basic solutions are employed for target molecules that exhibit poorly soluble in organic solvents in their neutral state. Acidic solutions, much like basic ones, drastically alter the protonation state of the target molecules by ionizing their functional groups. This strategically disrupts the non-covalent interactions, leading to a rapid loss of affinity for the polymer matrix [42,48].

Furthermore, proteins constitute a group of analytes that has garnered particular interest; however, their extraction remains challenging due to their large size and structural complexity [8]. Consequently, protein denaturation has emerged as an efficient technique for template removal. Five main approaches are commonly employed to remove this type of template: (i) denaturing agents such as SDS [49,50], and oxalic acid [51,52] (ii) high-salt solutions [53], (iii) acidic cleavage [54] (iv) alkaline treatment [55,56] and, (v) proteolytic treatment [57].

Although these methods facilitate the chemical separation of the template molecules from the polymer matrix, prolonged exposure to solvents induces swelling within MIP networks. These long immersion times can occasionally result in permanent topological changes. Therefore, it is crucial to use appropriate solvents that do not significantly swell or shrink the polymer, as well as optimizing extraction conditions such as exposure time, concentration, and pH. For instance, extreme pH values can lead to polymer hydrolysis, which drastically reduces the number of intact selective cavities, compromises the conductivity and electrochemical properties of the film, and consequently causes a notable decline in sensor performance [47,58].

**Figure 5:**
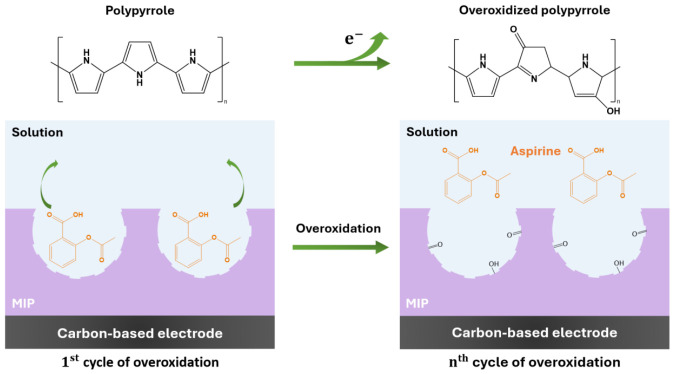
Overoxidation technique using CV to remove template molecules for cavity formation.

Overoxidation of conducting polymer: Unlike other techniques, template removal via overoxidation offers higher efficiency and mild removal conditions, preventing the MIP layer from swelling and thus retaining detection. One of the most widely used conductive polymers in the creation of sensors and biosensors is polypyrrole, due to its high chemical and thermal resistance, good biocompatibility, doping capacity, and electroactivity. An effective method for removing the template from the polymerized film derived from this type of conductive monomers is overoxidation [8]. This technique involves constantly exposing a polymer to a particular potential in a supporting electrolyte, without introducing any monomers or template molecules. As a result of this electrochemical reaction, carboxyl (–COOH), carbonyl (–C=O), and hydroxyl (–OH) groups are formed on the surface of the MIP (Figure 5). The generation of these groups decreases the MIP’s electrochemical characteristics, causing the template molecules and MIP to release their non-covalent bond [38]. Concurrently, overoxidation alters the π-conjugated backbone of the polymer, causing a notable decrease in conductivity and becoming the film electrically insulating, reflected in a strong in the charge transfer resistance (R_CT_). However, the simultaneous ejection of the template leaves behind empty cavities. These cavities function as localized charge-transfer pathways that facilitate electron transfer between the redox probe ions and the electron surface, leading to an increase in the electrochemical signal following overoxidation [59].

Previous studies have employed different electrochemical methods for polymer overoxidation. Chen et al. employed amperometry for tamoxifen removal from a polypyrrole-based MIP supported on carbon nanotubes, enabling its application as an electrochemical sensor to monitor tamoxifen in biological samples. The overoxidation was achieved by applying a constant potential (1.2 V) three times for 2 min in 0.1 M PBS [60]. Similarly, Wu et al. developed a MIP supported on MWCNT for the detection of the carcinogenic dye amaranth. The pyrrole was electropolymerized by cyclic voltammetry over the potential range of −0.20 to 0.80 V, and subsequent template removal was achieved amperometrically by applying at constant potential of 1.3 V twice for 120 s in 0.1 M PBS (pH 6.0) [61]. Alternatively, cyclic voltammetry can be used for overoxidation. For instance, Yulianti et al. demonstrated the overoxidation of polypyrrole by CV within a potential range of −0.7 to +1.0 V at a scan rate of 100 mV s^−1^ for 30 cycles, enabling the effective remove uric acid from the MIP cavities [38].

Even in electropolymerized MIPs, where film thickness can be controlled at the nanoscale, the high cross-linking density of conducting polymers can still entrap template molecules. This residual template can gradually leach out during sensing—a phenomenon known as template leakage. Such leakage generates unstable faradaic background currents, drastically worsening the sensor’s limit of detection and leading to false-positive outcomes. Ultimately, these limitations compromise the accuracy and reliability of the analytical results [47,62].

**Figure 6:**
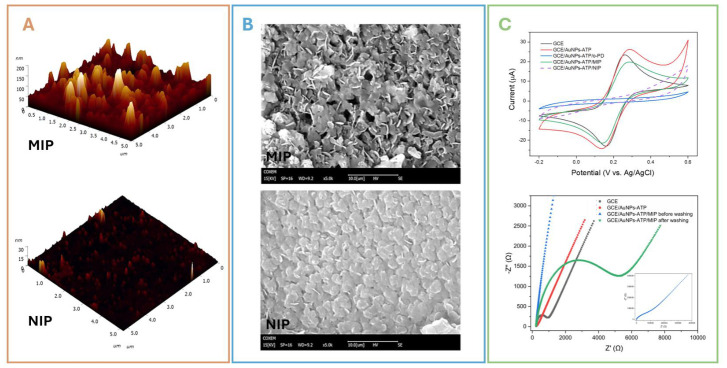
Physicochemical and electrochemical characterization of the sensor fabrication steps: (**A**) AFM topography images of the MIP and NIP. Adapted from ref. [63]. Copyright 2022, John Wiley and Sons; (**B**) SEM images of the MIP and NIP surfaces. Adapted from ref. [54]; (**C**) Cyclic voltammograms and EIS Nyquist plots of different fabrication stages. Adapted from ref. [64].

Although efficient template removal is critical for the optimal function of MIPs, it has received negligible scientific attention in comparison to all other imprinting steps. To ensure the absence of residual molecules that could trigger future leakage, the efficiency of this extraction process must be rigorously verified. Consequently, the efficiency of template removal is commonly verified by physicochemical and electrochemical techniques. For instance, Atomic Force Microscopy (AFM) is employed to evaluate changes in surface roughness, where an increase in surface roughness correlates with a more irregular and porous morphology, indicating the formation of available recognition cavities across the polymeric matrix after template elution. On the other hand, a decrease in roughness reflects a more compact and uniform surface, suggesting that these topographical defects are filled as target molecules rebind and occupy the cavities (Figure 6A) [63]. Scanning Electronic Microscopy (SEM) provides visual confirmation of template removal, revealing the appearance of cavities in the polymer structure. The micrographs indicate the absence of the template, confirming that imprinted sites are fully available for analyte interaction (Figure 6B) [54]. From an electrochemical perspective, electrochemical impedance spectroscopy (EIS) is widely used for monitoring this process. In the presence of a redox probe, such as potassium ferricyanide, template removal is typically evidenced by a decrease in the charge transfer resistance when comparing responses before and after template extraction (Figure 6C) [64].

In summary, while the electropolymerization of MIPs enables precise control over film thickness and recognition site formation, the technique presents certain limitations associated with the synthesis conditions and the physicochemical properties of the system. First, the electropolymerization step is strictly dependent on the pH of the medium, which restricts the working range and can compromise selectivity by altering the charge of the template. Furthermore, the chemical nature of the analyte represents a major challenge because the imprinting of electroactive templates can lead to their premature oxidation during the monomer electropolymerization, resulting in the formation of non-specific cavities (formed by oxidation subproducts). Finally, physical nature of the resulting material presents an additional challenge. Conducting polymers exhibit high hygroscopicity, altering their structure due to volume changes (such as swelling and collapsing), which introduce non-specific variations in electrical conductivity that affect the sensor’s reliability [65,66].

**Figure 7:**
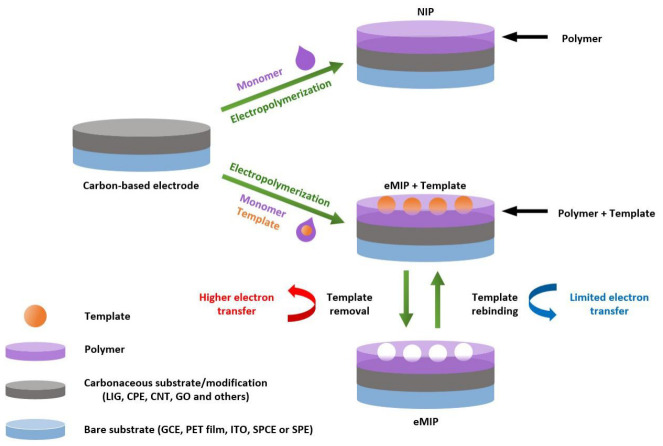
Schematic representation of the fabrication process and functional mechanism of electrochemical MIP sensors based on carbonaceous materials as supporting substrates. The upper pathway illustrates the formation of a non-imprinted polymer (NIP) as a control polymer, whereas the lower pathway depicts the eMIP process, including electropolymerization of the monomer–template mixture and the corresponding changes in electron transfer efficiency upon template binding and removal.

Beyond these polymer-specific limitations, the overall sensor performance is often further hindered by the intrinsic properties of the bare electrode. To address these combined challenges and the growing demand for ultra-sensitive diagnostics with low detection limits, various nanomaterials have been explored as surface modifiers. Among these, carbonaceous materials—ranging from carbon nanotubes (CNTs) to graphene and carbon quantum dots (CQDs)—have emerged as promising supporting substrates (Figure 7). These materials offer enhanced electronic properties, high surface-to-volume ratios, and rapid electron transfer kinetics, leading to significantly lower limits of detection (LODs) [67]. Ultimately, the integration of these carbon platforms with eMIPs creates a synergistic effect, combining the tailored selectivity of molecularly imprinted cavities with the excellent conductivity and catalytic activity of carbon nanomaterials [68].

## 3. Carbon-Based Materials as Supporting Substrates on eMIPs

In electropolymerized molecularly imprinted polymer (eMIP)–based sensors, carbon materials are widely used as supporting substrates for detecting pharmaceuticals (e.g., paracetamol, ibuprofen, ramipril) [69], biomolecules (e.g., glucose, dopamine) [70], and environmental contaminants (e.g., pesticides, heavy metals) [40]. Traditionally, glassy carbon (GC) has served as the fundamental pure carbon platform for these electrochemical applications [10]. Similar in structure to amorphous carbon, GC is composed predominantly of sp^2^-hybridized carbon atoms arranged in a non-graphitizing structure. It consists of small, disordered graphite-like domains with partly stacked but mismatched units, whose edges are terminated by hydrogen or hydroxyl groups [71]. This unique configuration provides high electrical conductivity, electrochemical inertness over a broad potential window, chemical stability, and ease of surface modification [72].

Despite these foundational advantages, bare GCEs present certain analytical and practical limitations. They typically require pretreatments, such as electrochemical activation or mechanical polishing, to achieve optimal electrocatalytic activity [73]. More importantly, their rigid, macroscopic nature restricts their use in modern applications demanding portability, and their intrinsic electrochemically active surface area is often insufficient for ultra-low trace detection. Consequently, bare GCEs frequently require surface modification with additional nanostructured materials to expand their active area and improve their selectivity toward specific analytes [74].

To overcome these geometric constraints and meet the surging demand for point-of-care diagnostics, portable environmental monitors, and flexible wearable devices, research over the past five years has strategically shifted toward more advanced, scalable, and cost-effective carbonaceous alternatives. This transition has driven the exploration of diverse multidimensional carbon substrates, including carbon nanotubes (CNTs), graphene, zero-dimensional dots (CQDs, NCDs), and macroscopic flexible platforms like carbon cloth (CC) and biomass-derived carbon. Whether utilized as standalone flexible electrodes or as nanostructured modifiers to upgrade a traditional GCE, these materials effectively address previous limitations by maximizing active surface areas, enabling tunable surface chemistry, and providing exceptional structural flexibility without compromising conductivity.

In these advanced architectures, the carbonaceous nanostructures not only provide robust mechanical support for polymer growth but also act as highly efficient conductive pathways, facilitating rapid electron transport between the recognition sites and the electrode surface [75,76]. This synergistic interaction between the emerging carbon materials and the imprinted polymer network directly enhances sensitivity, accelerates response times, and improves overall signal stability [41]. As a result, these systems often outperform traditional MIP sensors in terms of detection limits, with some achieving ultra-trace sensitivity for analytes such as pesticides in complex matrices, including wastewater [77]. Accordingly, this section discusses the main classes of carbon-based materials currently employed in eMIP fabrication, highlighting their structural characteristics, economic and operational advantages, and emerging trends in the development of next-generation electrochemical (bio)sensors.

### 3.1. Carbon Nanotubes

The integration of carbon nanotubes (CNTs) with molecularly imprinted polymers has become one of the most extensively explored strategies for enhancing the performance of electrochemical sensors [77]. Owing to their high electrical conductivity, large specific surface area, and excellent mechanical stability, CNTs facilitate rapid electron transfer and provide a favorable interface for the growth of electropolymerized MIP films [43]. Both single-walled (SWCNTs) and multi-walled carbon nanotubes (MWCNTs) have been employed as electrode modifiers, with surface functionalization frequently used to improve dispersion, increase the density of anchoring sites, and promote stronger interactions with the imprinted polymer matrix [78]. In addition to improving signal transduction, CNTs contribute to the formation of thinner and more homogeneous imprinted films, enhancing analyte accessibility to recognition sites and reducing diffusion limitations [79].

While pristine CNTs are chemically inert, their effective integration into eMIP platforms typically requires prior chemical modification [80]. Functionalization, either covalent (e.g., carboxylation, hydroxylation, amination) or non-covalent (e.g., polymer wrapping), introduces reactive surface sites that enhance dispersibility, improve compatibility with polymer matrices, and promote more efficient electron transfer [36,45,60]. Such chemical tuning of SWCNTs, MWCNTs, and nitrogen-doped nanotubes (NCNTs) plays a crucial role in maximizing sensor performance [38]. Specifically, SWCNTs offer excellent signal transduction due to their superior electrical properties, whereas MWCNTs provide greater mechanical robustness and a larger surface area for polymer attachment [61]. In particular, MWCNTs can be further engineered through controlled synthesis and chemical treatments that introduce structural defects, thereby enhancing sensor performance [81]. However, variability in CNT size, chirality, and surface chemistry between batches—even for carboxylated MWCNTs—can significantly affect the reproducibility of eMIP fabrication and sensor responses [82]. This heterogeneity remains a major challenge in translating lab-scale sensors into reliable, commercial point-of-care diagnostic platforms [83].

To overcome individual limitations and further expand their applicability, recent developments have focused on combining chemically modified CNTs with other nanomaterials to form high-performance composites. For instance, integrating transition metals (e.g., nickel nanoclusters) or noble metal nanoparticles (e.g., gold) significantly improves sensitivity by enhancing electrocatalytic activity and facilitating analyte access to the imprinted sites [52,76,84]. Furthermore, incorporating semiconductor transition metal oxides, such as ZnO, creates synergistic heterojunctions with the carbon network. This not only enhances overall chemical stability but also accelerates interfacial electron-transfer kinetics for superior catalytic performance [84]. Beyond signal amplification, these metallic components reinforce the typically brittle polymeric matrix of MIPs. This synergistic combination—compatible with a wide range of functional monomers (e.g., pyrrole, thiophenol, and o-phenylenediamine)—leads to improved durability, stronger adhesion to the electrode surface, and increased resistance to environmental variations (e.g., pH and ionic strength), all of which are essential for robust in real-sample analysis [85].

Regarding sensor fabrication, electropolymerization enables the direct formation of uniform and compact MIP films on CNT-modified electrodes, providing precise control over film thickness and morphology. For example, in a recently reported CNFs/CNTs aerogel-based cortisol sensor, the porous three-dimensional carbon network promoted efficient mass transfer and enhanced accessibility of the imprinted cavities, contributing to improved sensing performance [75]. However, careful optimization of the electropolymerization conditions remains essential. Excessive polymer growth may partially cover the conductive nanocarbon surface, reduce the availability of recognition sites, and hinder analyte diffusion [37]. These effects become more pronounced in dense CNT assemblies or overly thick MIP layers, ultimately leading to diminished sensitivity and slower sensor response [86].

Despite these fabrication challenges, the highly tunable properties of CNT-MIP sensors have enabled their widespread application across diverse analytical fields. In environmental monitoring, they are employed for the detection of persistent pollutants in complex matrices such as tap water, wastewater, and landfill leachate, consistently achieving low detection limits [87]. In biomedical applications, these platforms allow highly sensitive detection of key biomarkers, including thrombin, dopamine, uric acid, and growth hormone-releasing hexapeptide [88]. Furthermore, CNT-MIP sensors have demonstrated strong performance in pharmaceutical analysis, reliably detecting compounds like chlortetracycline in real samples (e.g., lake water, milk, and pork) with excellent recovery rates ranging from 86.4% to 112.0% [89].

Ultimately, carbon nanotubes are frequently preferred over graphene and carbon black for MIP sensor modification due to their superior electrical conductivity within 3D networks, providing higher electron mobility, mechanical flexibility, and easier functionalization [2]. Nevertheless, their intrinsic one-dimensional structure tends to promote aggregation, a challenge that reduces the accessible surface area and limits sensor performance [70]. To fully exploit the potential of CNTs, achieve proper dispersion and alignment is crucial, as this maximizes active surface interactions and enhances overall efficiency [90]. As ongoing research addresses these aggregation challenges, CNT-MIP composites will continue to drive significant advances in electrochemical sensing, particularly regarding molecular recognition, signal transduction, and integrated device architectures.

### 3.2. Graphene

**Figure 8:**
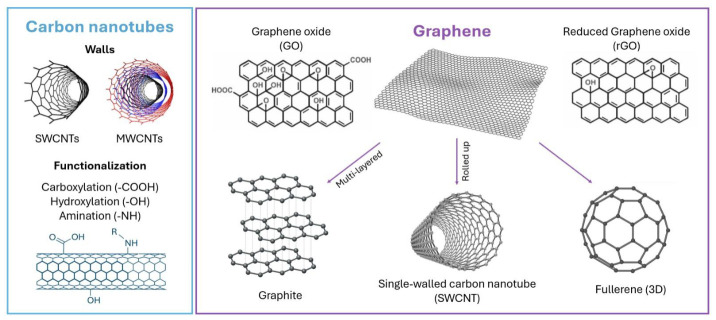
Main carbon-based substrates for eMIP sensors. The left panel shows the structure and common covalent functionalization of carbon nanotubes, while the right panel illustrates graphene as a building block for other carbon allotropes and its oxidized derivatives (GO and rGO).

Among carbonaceous materials, graphene is the second most widely utilized substrate for eMIP sensors. Fundamentally, graphene consists of a single layer of sp^2^-hybridized carbon atoms arranged in a two-dimensional (2D) honeycomb lattice, serving as the basic building block for several other carbon allotropes (Figure 8). Its highly delocalized π electron system provides exceptional electrical conductivity, while its conjugated structure facilitates π-π stacking interactions with aromatic or unsaturated template molecules—a critical feature for enhancing selective recognition [14,91]. Consequently, the integration of graphene and its versatile derivatives (such as graphene oxide, reduced graphene oxide, laser-induced graphene, and their composites) with electropolymerized recognition layers has fueled a new wave of ultra-sensitive, stable, and highly selective eMIP sensors for a broad spectrum of analytes [54,92].

Graphene oxide (GO) and reduced graphene oxide (rGO) are the most studied derivatives, differing primarily in their oxygen content and resulting physicochemical properties. GO is rich in oxygen-containing functional groups—such as hydroxyl, epoxy, and carboxyl groups—which disrupt the continuous sp^2^ carbon network but simultaneously provide ideal reactive sites for further covalent functionalization and polymer anchoring [93]. For instance, carboxylated GO (GO-COOH) features an expanded interlayer spacing and abundant anchoring points, making it a highly efficient platform for MIP sensors [94]. Through advanced electropolymerization techniques, GO-based platforms have successfully pushed detection limits down to pico- and nanomolar levels for diverse target molecules, including cholesterol, dopamine, and benzene [95].

To further enhance sensor performance, the design of imprinted matrices containing rGO frequently incorporates various metallic nanoparticles to boost electrocatalytic activity and surface area. In the literature, rGO has been successfully combined with decorated manganese nanoparticles for palmitic acid detection in guava seed oil [96]. Furthermore, integrating rGO with bimetallic transition metal oxides, such as NiCo_2_O_4_ nanoparticles, takes advantage of synergistic multiple oxidation states to significantly amplify the signal for diagnosis of polycystic ovary syndrome via follicle-stimulating hormone detection [97]. Similarly, the incorporation of gold nanoparticles (AuNPs) with rGO nanoribbons has enabled the highly sensitive and selective detection of mycotoxin in food samples [98]. Beyond clinical and food safety analyses, MIP-rGO nanocomposites are also extensively applied in environmental monitoring, particularly for the quantification of agricultural pesticides and herbicides [24].

In recent years, the drive toward portable and wearable sensors have led to the widespread adoption of Laser-Induced Graphene (LIG) and carbonized paper electrodes. LIG is a highly porous 3D graphene material generated by exposing carbon-rich precursors, such as polyimide films, to a targeted laser beam, rapidly transforming the surface into a conductive network without requiring complex fabrication steps [99]. Leveraging this approach, Animashaun et al. developed an enzyme-free LIG-MIP biosensor for lactate detection in artificial saliva (LOD = 0.033 μM) by synthesizing the MIP directly on the LIG surface, they create a cost-effective and portable tool suited for on-site clinical screening [100].

Parallel to polyimide substrates, laser-induced carbonization and pyrolysis have also been innovatively applied to paper matrices. Composed of cellulose fibers, these modified paper substrates provide a conductive, cost-effective, and highly flexible platform for microfluidics. A notable application involves a paper-based microfluidic wearable sensor that integrates a MIP platform to monitor sweat cortisol levels via electrochemical impedance spectroscopy (EIS). This sophisticated device dynamically induces sweat via iontophoresis while simultaneously quantifying sweat volume, secretion rate, sodium ions, and cortisol concentrations [99]. Together, these carbonaceous materials highlight the vast potential of graphene-derived substrates in driving the next generation of point-of-care diagnostics and advanced biosensing architectures.

### 3.3. Other Carbon Materials

**Figure 9:**
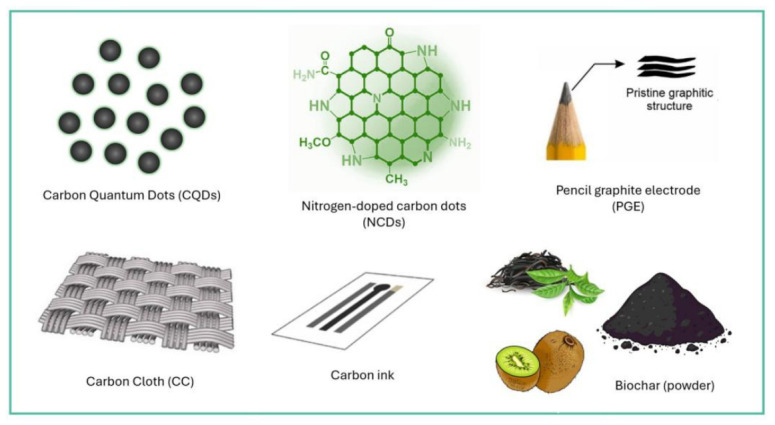
Diverse carbon-based substrates for eMIP sensor fabrication, highlighting nanoscale modifiers (CQDs and NCDs), rigid and cost-effective electrodes (PGE), flexible macroscopic supports (CC), printable technologies (carbon ink), and sustainable biomass-derived carbon (biochar).

In the past five years, several advanced carbonaceous materials (Figure 9)—beyond conventional carbon nanotubes and graphene derivatives—have been explored as surface modifiers for electropolymerized MIP sensors to improve electron transfer, sensitivity, and selectivity. Besides zero-dimensional materials such as carbon quantum dots (CQDs) and nitrogen-doped carbon dots (NCDs), growing attention has been directed toward aerogel composites, biomass-derived porous carbons [101], and carbon hybrid electrodes [15]. These emerging materials offer complementary advantages, including high surface area, hierarchical porosity, enhanced conductivity, and scalable fabrication strategies, which favor efficient electropolymerization and improve analyte accessibility to the imprinted recognition sites. In particular, carbon-based nanohybrids combine distinct nanomaterials with complementary physicochemical properties, generating synergistic effects that enhance charge-transfer kinetics, mass transport, and overall sensor performance beyond those achievable with the individual components alone.

In this context, Lu et al. developed an electropolymerized MIP sensor based on AuNPs and nitrogen-doped graphene oxide quantum dots supported on a NiS_2_/biomass-derived carbon composite [102]. The biomass-derived carbon provided a porous and conductive framework, while the incorporation of quantum dots and AuNPs enhanced electron-transfer kinetics and electrocatalytic activity. Similarly, a disposable eMIP sensor for amyloid-β42 was fabricated using a nitrogen-doped carbon dot/graphene nanohybrid deposited on a screen-printed carbon electrode [103]. In this case, the synergistic combination of carbon dots and graphene increased the electroactive surface area and facilitated charge transport, resulting in highly sensitive biomarker detection. These studies illustrate the growing use of carbon-based nanohybrids and biomass-derived materials as advanced supports for eMIPs, where complementary structural and electrochemical properties contribute to improved sensing performance.

Aligning with the trend of sustainable analytical chemistry, biomass-derived carbon has emerged as a promising substrate. Tea branch biochar, a porous material derived from the pyrolysis of agricultural waste, exemplifies this approach. Recently, potassium carbonate-activated tea branch biochar was utilized to coat a MIP sensor for norfloxacin detection. Its unique porous structure, high surface area, and abundance of oxygen-containing functional groups improved electrical conductivity and provided dense active sites for polymer growth. This integration significantly enhanced sensor performance in complex matrices like milk, honey, and pork meat [48]. Ultimately, incorporating such biomass into MIP platforms not only advances green chemistry in sensor fabrication but also contributes to agricultural waste valorization.

Transitioning to macroscopic flexible supports, porous carbon cloth (CC) and carbon fiber paper (CFP) are increasingly utilized for their well-defined porous structures, large surface areas, and high conductivity, which collectively foster efficient electron transfer and analyte accessibility. For instance, a novel sensor for perphenazine detection was constructed by electrodepositing a Cu-coordinated MIP onto a Ag nanoparticle-modified flexible carbon cloth [104]. Similarly, MnCO_3_ nanostructures were incorporated into carbon fiber cloth (CFC) to produce MIP sensors for determining ochratoxin A in apple juice [53]. Complementing these textile-like substrates, CFP serves as an ideal scaffold for nanomaterial immobilization. George et al. paired CFP with high-surface-area graphitic carbon-nitride (gCN) to develop a highly efficient MIP platform for measuring trace amounts of butylated hydroxyanisole, a crucial antioxidant in food safety [105].

The structural versatility of these diverse carbon sources—ranging from graphite and carbon black to biochar and nanotubes—has also driven the development of conductive carbon inks. These inks are foundational for fabricating novel flexible electrodes. By providing the necessary electrical conductivity and seamlessly integrating with MIP technology, carbon inks enhance signal transduction in highly specialized formats. Consequently, the incorporation of these printable carbon substrates facilitates the creation of biodegradable, biocompatible, and personalized diagnostic tools, including wearables, implants, and tissue-like digestible sensors [52,106].

Finally, to address practical challenges related to sensor longevity and reusability, pencil graphite electrodes (PGEs) have emerged as a highly cost-effective and renewable alternative. Unlike other carbonaceous materials that suffer from irreversible surface fouling due to the adherence of analytes or reaction by-products, PGEs allow for rapid and economical surface renewal. Demonstrating their robustness, El Azab et al. developed a selective PGE modified with AuNPs and a molecularly imprinted levodopa polymer to quantify empagliflozin (a type 2 diabetes medication). Notably, this platform enabled direct measurements in tablets, human plasma, and urine samples without the need for prior extraction, achieving impressive recovery rates between 95.78% and 100.80% [107].

## 4. Applications in (Bio)Sensing

The various molecularly imprinted polymers obtained by electropolymerization on carbon-based materials have enabled the development of highly selective sensors and biosensors. These platforms can be applied to a wide range of analytes, relevant to clinical diagnostics (e.g., CRP, albumin, glucose, dopamine, uric acid) and environmental monitoring (e.g., pharmaceutical residues, industrial dyes) [69].

To provide a comprehensive bibliometric overview of the current state of the art, a quantitative analysis of the compiled sensing systems from the 55 reviewed studies (summarized in the following subsections) was performed. Regarding functional monomers, pyrrole (*n* = 16) and *o*-phenylenediamine (*n* = 14) emerge as the most widely utilized building blocks for eMIP fabrication onto carbon-based materials, followed by fewer reports on dopamine, *o*-aminophenol, and acrylates, among others. In terms of advanced carbon-based supports, variations in CNTs (*n* = 26) and GO (*n* = 13) clearly dominate the literature, being deployed either as single-component modifiers or combined into hybrid carbonaceous nanocomposites. Additionally, emerging or alternative platforms—such as carbon fibers, biomass-derived biochar, LIG, PGE, CPE, and graphene derivatives—have also been successfully explored to upgrade traditional interfaces.

Regarding electroanalytical transduction, DPV is unequivocally the preferred method (*n* = 42), significantly outpacing SWV, electrochemical impedance spectroscopy (EIS), amperometry, CV, and linear sweep voltammetry (LSV). These architectures have been applied across diverse analyte categories, primarily focusing on drugs and pharmaceuticals (*n* = 23), biological compounds (*n* = 13), environmental pollutants (*n* = 11), proteins (*n* = 7), food security targets (*n* = 3), and industrial dyes (*n* = 2), including advanced dual-sensor configurations designed for simultaneous multi-analyte quantification. The engineered sensors demonstrate remarkable analytical performance, with LOD spanning from mM up to an ultra-trace level of fM. Crucially, in alignment with the analytical shortcomings discussed hereafter, only 34.5% (*n* = 19) of these published works explicitly report the Imprinting Factor (IF), and a striking minority of merely 3.6% (*n* = 2) provide a rigorously calculated Selectivity Factor (SF), reinforcing the urgent need for methodological and data standardization in the field.

Beyond these bibliometric trends, to achieve optimal quantification of these targets, eMIP sensing architectures typically operate through distinct mechanisms—namely, direct and indirect detection strategies. Consequently, the selection of the appropriate electroanalytical technique (e.g., DPV, SWV, CV, amperometry, or EIS) is not universal, but rather highly dependent on the chosen sensing mechanism and the nature of the target analyte. The following subsections detail these direct and indirect detection strategies, provide a focused guideline for optimal technique selection, and conclude with a critical evaluation of the analytical reporting practices in current eMIP literature.

### 4.1. Direct Detection: Electroactive Template

**Figure 10:**
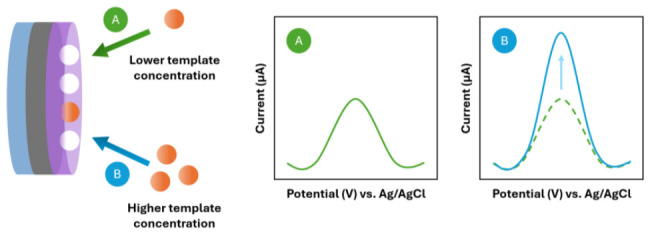
Direct detection in a MIP where the template (analyte) generates a redox process and the current peak increases when occupying more cavities, i.e., the electrochemical signal increases with the concentration of the analyte.

Many of the analytes studied by electrochemical sensors are electrochemically active (Table 2), meaning they exhibit characteristic oxidation or reduction peaks when analyzed in an adequated supporting electrolyte. Therefore, using electroactive analytes as templates in the formation of MIPs can lead to direct detection, where the recorded electrochemical signal is due to the oxidation or reduction in the analyte itself. In this type of detection, the rebinding of the target analyte in the selective cavities of the MIP generates an electrochemical response characteristic of that specific molecule. Thus, as the template concentration increases, the polymer cavities fill and the peak current increases (Figure 10).

#### 4.1.1. Drugs and Pharmaceuticals

The widespread and often indiscriminate consumption of antibiotics, analgesics, and anti-inflammatory agents underscores the urgent need for versatile analytical monitoring tools. In this context, a dual sensor was recently developed for the simultaneous determination of ethionamide (ETH), an antituberculosis drug, and antipyrine (AnP), an analgesic and anti-inflammatory agent. The device was based on a reduced graphene oxide (rGO) nanocomposite modified with an electropolymerized poly(3-thiopheneacetic acid) (TAA) film. A major advantage of this dual-template system lies in the distinct electroactivity of the two analytes, enabling the detection of the ETH signal at −1.4 V and the AnP signal at +1.25 V. Owing to this wide peak potential separation, the sensor achieved a LOD of 0.15 μM and 0.117 μM, respectively, demonstrating excellent selectivity and resistance to matrix effects in complex biological fluids [63].

The growing consumption of anticancer drugs demands rigorous monitoring, as their severe cytotoxicity and environmental persistence threaten both human health and aquatic ecosystems. To address this, carbon-supported eMIPs provide the sensitivity and selectivity required to quantify these compounds at trace levels in complex matrices, such as biological fluids and hospital effluents. A notable example is tamoxifen, a widely prescribed breast cancer drug. For its determination, Chen et al. developed an eMIP sensor using CNTs as support, which provided greater stability and binding capacity. Consequently, this system achieved a LOD of 11.3 nM in biological matrices, outperforming conventional eMIP sensors [60].

**Figure 11:**
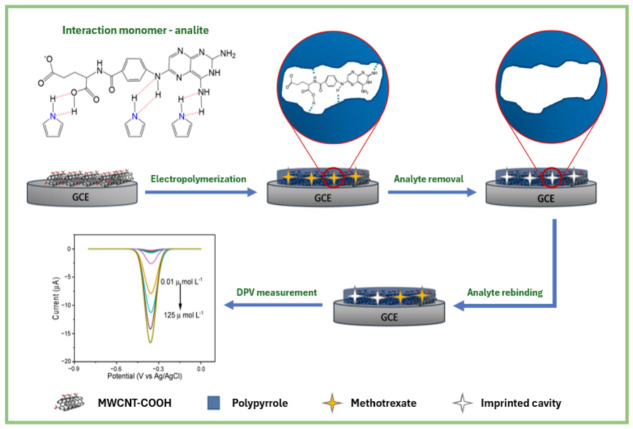
Schematic illustration of the preparation of electropolymerized MIP film and its application for the quantification of methotrexate. Adapted from ref. [45].

Similarly, Jara-Cornejo et al. fabricated a sensor based on carboxyl-functionalized MWCNTs (-COOH) and polypyrrole film for methotrexate detection. The inclusion of the carbonaceous material amplified the faradaic current response up to threefold compared to the bare glassy carbon electrode (GCE). Meanwhile, the formation of selective cavities was possible due to hydrogen bonding interactions between the amine and carboxyl groups of methotrexate and the amine group of pyrrole. Template extraction was performed at pH 10 via polypyrrole overoxidation (+0.3 to +1.0 V). This process imparted negative charges to both the sensor and methotrexate, inducing the drug’s release through electrostatic repulsion. Unlike typical oxidation-based methods, quantification was performed by monitoring the reduction signal of methotrexate using DPV (Figure 11). The sensor exhibited excellent performance, demonstrating high selectivity, repeatability (RSD = 2.50%), and reproducibility (RSD = 1.72%). Furthermore, it was successfully applied to environmental and pharmaceutical samples, yielding recoveries between 102% and 105% [45].

Monitoring antipsychotics is crucial due to their severe side effects and environmental risks. Although conventional electrochemical sensors are sensitive and cost-effective, their reproducibility is often compromised by external factors. To address this, ratiometric sensors have emerged, which significantly improve analytical robustness by measuring the ratio between the analyte’s signal and an electroactive internal reference. In this context, Liu et al. developed a ratiometric sensor for perphenazine (PPZ) detection using a porous carbon cloth (CC) substrate modified with silver nanoparticles (AgNPs) to enhance electrocatalytic activity. A copper-coordinated polymer film was electropolymerized onto the electrode to increase adsorptive capacity. The peak current ratio between PPZ and the AgNPs exhibited a linear response to the PPZ concentration ranging from 1 to 700 nM, achieving a LOD of 0.43 nM. The sensor demonstrated high selectivity, sensitivity, reproducibility, and excellent anti-interference capabilities. The practicability of the sensor was evaluated in human serum and pharmaceutical samples, yielding satisfactory recoveries ranging from 92.46% to 104.90% [104]. Similarly, Yuan et al. reported a ratiometric sensor for chlorpromazine (CPZ) using copper-coordinated polydopamine, which acts as both a polymer matrix and an internal reference probe. This system was integrated with a cost-effective and catalytic composite of bimetallic oxide (Zn/Mn) and MWCNTs, which improves the dispersion of nanoparticles. The Cu-MIP/ZMO/MWCNTs/GCE sensor exhibited a linear range from 1 nM to 10 μM and a LOD of 0.42 nM. Its application in human serum and lake water samples showed satisfactory recoveries (85.0–116.0%), comparable to HPLC analysis [86].

Shifting to antibiotics, Niu et al. developed an electrochemical sensor to detect sulfamethazine, an antibiotic with hepatotoxic and nephrotoxic effects. The device employs a polydopamine MIP film supported on a CNTs/MoS_2_-NiCo composite. The template-to-monomer ratio was optimized using density functional theory (DFT) calculations to maximize stability and the formation of selective cavities. The sensor achieved a LOD of 0.033 μM and a broad linear range (0.1–800 μM). When tested in meat samples, it showed satisfactory recoveries (98–104%) and demonstrated high reproducibility, repeatability, and stability [108].

**Figure 12:**
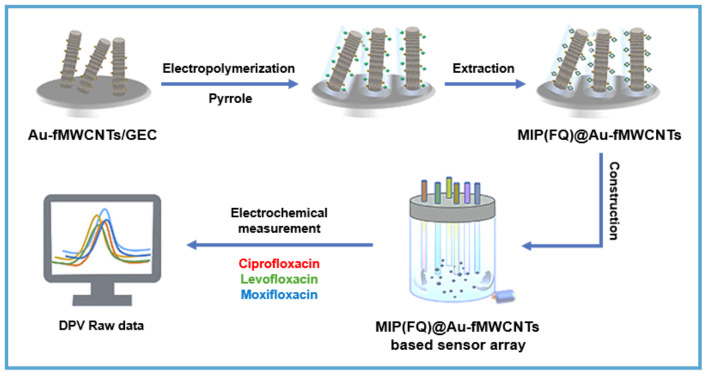
Schematic illustration of the preparation of sensor array based on MIP for the analysis of fluoroquinolone antibiotics. Adapted from ref. [109].

Another group of antibiotics widely used in industry are fluoroquinolones. However, their excessive use has led to contamination issues and antimicrobial resistance. Therefore, Wang et al. developed a voltammetric electronic tongue for the detection of this family of drug. Their device consisted of three MIPs obtained by pyrrole electropolymerization, and integrating MWCNTs decorated with gold nanoparticles, combining the high surface/mass ratio of the CNTs, with the high conductivity and the catalytic effect of the AuNPs. Figure 12 shows the MIP film formed along the surface of the Au-fMWCNTs, allowing for better control over the film’s distribution and thickness. This sensor was capable of detecting fluoroquinolone concentrations within a linear range of 2 to 300 µM by DPV in a potential range from +0.6 to +1.4 V. Through the use of chemometrics, the sensor achieved the simultaneous quantification of ciprofloxacin, levofloxacin, and moxifloxacin; furthermore, it exhibited good selectivity against other families of drugs [109].

#### 4.1.2. Environmental Pollutants

**Figure 13:**
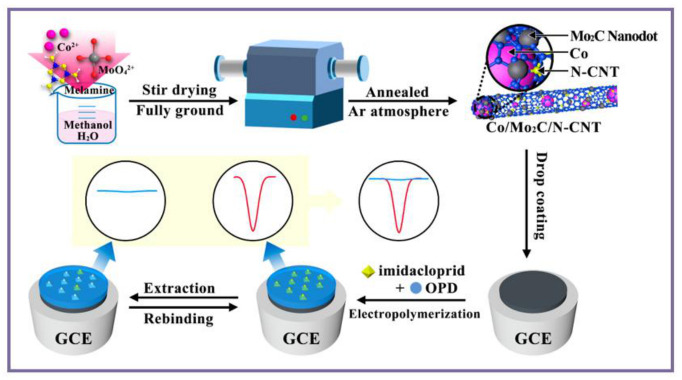
Schematic illustration of the fabrication of the electrochemical sensor based on Co/Mo_2_C/N-CNT composite material for imidacloprid detection. The resulting sensor exhibits a distinct electrochemical reduction response upon capturing the target analyte. The blue and red curves represent the pulse voltammetry responses after template extraction (no reduction peak) and after imidacloprid rebinding (catalytic current peak), respectively. Reproduced from ref. [110].

Pesticides are one of the main groups of environmental pollutants. Although they are widely used in agriculture, their excessive use causes degradation of aquatic and terrestrial ecosystems, posing a toxicological risk to humans via food contamination. One of these pesticides is zineb, which cause neurodegenerative disorders. Therefore, Mahmodnezhad et al. developed an electrochemical sensor based on SWCNTs for zineb detection, integrating a MIP synthesized via the electropolymerization of *o*-aminophenol. The synergy between the carbonaceous material and the MIP provided excellent sensitivity and selectivity, enabling the quantification of the pesticide within a linear range of 5 to 1000 fM, and a LOD of 1.6 fM [42]. Another relevant pesticide is imidacloprid (IMI). While it exhibits low toxicity, high efficiency, and a long duration of action, its environmental accumulation has adversely affected human health, pollinating insects, and fish. In this way, Feng et al. developed a Mo-Mo_2_C catalyst anchored on N-CNTs to fabricate an electrochemical MIP-based sensor for the detection of IMI (Figure 13). The composite (Co/Mo_2_C/N-CNT) enhanced the device’s sensitivity due to the abundant availability of active sites, improved catalytic performance, and efficient electron transfer. Furthermore, this nanomaterial facilitated the adsorption of the nitro and C=C groups of IMI, an essential step for the proper formation of selective cavities. DPV was employed for quantification by monitoring the analyte’s reduction over a linear range of 0.1 to 100 µM, achieving a LOD of 0.033 µM. Finally, the method’s stability was confirmed over two weeks (96.3% of retention), and it was successfully applied for the in situ analysis of tea samples [110].

Beyond their environmental impact, industrial dyes represent a significant risk to human health. Although their concentrations in consumer products are strictly regulated, non-compliance with these limits in the food industry causes harmful effects: excessive consumption is closely linked to conditions such as asthma, cancer, allergic reactions, and anxiety disorders. Consequently, their selective quantification in complex food matrices is imperative. A representative case is amaranth, an azo dye responsible for the red hue in cakes, beverages, and desserts, whose acceptable daily intake established by the WHO (World Health Organization) is 0.5 mg/kg. For its monitoring, an eMIP sensor based on a polypyrrole supported on MWCNTs has been reported. This nanocomposite successfully generated selective recognition cavities, promoted by the synergy of electrostatic interactions and strong hydrogen bonds. During the synthesis stage, these interactions are specifically established between the sulfoxide (S=O) or hydroxyl (-OH) groups of amaranth and the amine (-NH) groups of pyrrole [61].

Similarly, tartrazine is another widely used dye in the food industry, responsible for imparting yellow color. For its determination, a sensor was developed using a carbon paste electrode (CPE) as a cost-effective alternative to the conventional glassy carbon electrode (GCE). The recognition mechanism of this MIP exploited interactions analogous to the previous case, relying on the affinity between the sulfoxide and hydroxyl groups of the analyte and the amine groups of the functional monomer, which in this specific design was arginine [111].

#### 4.1.3. Biological Compounds

Biological compounds are responsible for regulating fundamental physiological processes, and maintaining their levels within strict ranges is critical for homeostasis. Consequently, the accurate quantification of these species in biological fluids is essential for the early diagnosis of various pathologies. Although macromolecules often present limitations for direct electron transfer, there is a broad group of low-molecular-weight biomolecules that are electroactive. These species can be determined via direct electrochemical techniques, exploiting their ability to undergo oxidation or reduction at specific potentials, which enables the development of rapid, label-free sensors [70,102].

**Figure 14:**
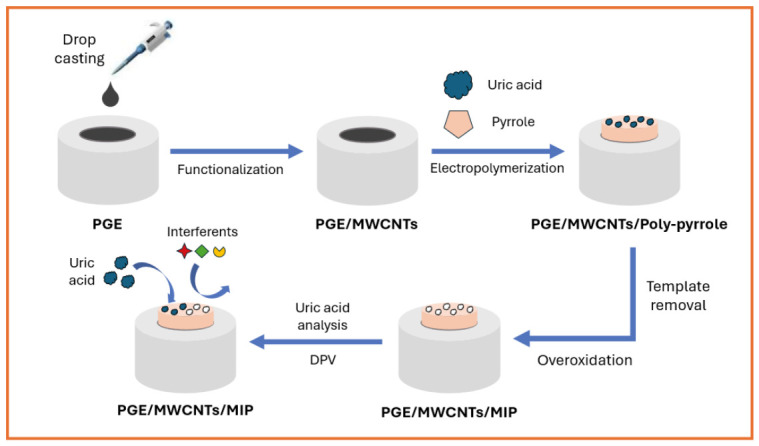
Schematic illustration of the electrochemical sensor fabrication based on PGE/MWCNTs/MIP for uric acid detection. Redrawn from ref. [38].

Demonstrating this principle, Yulianti et al. developed a uric acid sensor using a MIP that simulated the biological mechanism of uricase. This was achieved by depositing MWCNTs onto a commercial pencil graphite electrode (PGE), followed by the electropolymerization of pyrrole (Figure 14). The analytical performance of the eMIP was evaluated in a uric acid solution via DPV, exhibiting a linear range of 0.22 to 3.5 mM and a LOD of 0.76 mM. Furthermore, the sensor demonstrated a remarkable sensitivity of 97.459 µA µM^−1^ cm^−2^ and retained 71% of its initial stability after 19 days of use [38].

Among electroactive neurotransmitters, dopamine is particularly relevant due to its clinical applications, as its fluctuations are associated with several neurological disorders. While classical analytical techniques, such as chromatography and spectroscopy, achieve trace-level detection, their high costs and complex instrumentation limit their in situ applicability. In contrast, electrochemical sensors represent a viable alternative for the development of miniaturized devices. In this context, Nekoueian et al. fabricated an eMIP sensor using a polypyrrole nanocomposite electropolymerized onto carbon nanofibers (CNFs). The incorporation of CNFs generated a synergistic effect that not only increased the effective surface area and enhanced the accessibility of the imprinted sites, but also accelerated electron transfer kinetics due to the highly conductive carbonaceous network. This system enabled the quantification of dopamine in cell culture media via DPV, achieving a LOD of 62.57 nM with remarkable selectivity against coexisting biological interferents [70].

In vivo analysis using electrochemical sensors has emerged as a novel quantification strategy that avoids time-consuming sample treatment and simplifies the analytical procedure. Lin et al. developed a sensor for the in vivo analysis of indole-3-acetic acid (IAA) in tomato, a key phytohormone regulating plant growth and development. For the detection of trace amounts of IAA, the authors fabricated eMIPs on a carbon ink screen-printed PET surface via the electropolymerization of pyrrole. Furthermore, this sensor was coupled with a hydrogel for non-invasive analysis in tomato leaves, achieving LOD of 0.1 µM [106]. In a related study, Tian et al. continued exploring this analyte by developing an eMIP. The working electrode consisted of a stainless-steel rod modified with carbon ink and coated with an epoxy resin (AB glue). The final needle-shaped sensor was inserted for the in vivo analysis of the ripening level in tomatoes, demonstrating reliable responsiveness even in the presence of interferents such as tryptophan [112].

### 4.2. Indirect Detection

**Figure 15:**
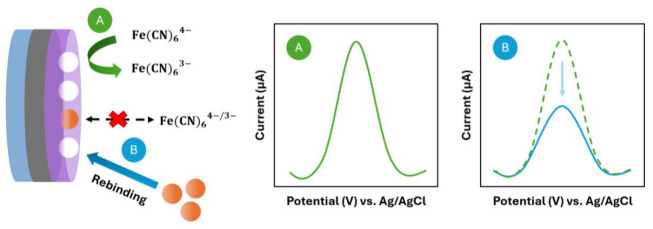
Indirect detection, where the template blocks the redox probe processes (such as ferro/ferricyanide or hexaammineruthenium solutions), leading to a decrease in the peak current.

In indirect sensing strategies (Table 3), the electrochemical response typically exhibits an inverse relationship with the analyte concentration. Depending on the sensor architecture, this detection mechanism can rely on either the modification of faradaic reduction/oxidation properties of a probe, or the alteration of the intrinsic conductivity and capacitive behavior of the sensing material itself. The most common approach employs a soluble redox probe (typically [Fe(CN)_6_]^3−/4−^ or [Ru(NH_3_)_6_]^3+/2+^). As the concentration of the target species increases, a pronounced decrease in the redox peak current is observed (Figure 15) [89]. This signal attenuation arises from the progressive occupation of the imprinted recognition sites by the analyte molecules, which effectively act as physical and electrochemical barriers. Once bound within the cavities, these molecules hinder the diffusion of the redox probe toward the electrode surface and partially block active sites, suppressing interfacial electron transfer between the solution-phase redox probe and the underlying carbon electrode surface electrode [51]. Consequently, the extent of current suppression can be directly correlated with the degree of site occupancy, enabling quantitative analysis through signal inhibition mechanisms.

However, relying on a soluble redox probe imposes significant constraints on sensor design and applicability. From a design perspective, the continuous requirement to add a soluble reagent limits the transition of these sensors into reagentless, portable point-of-care devices. Regarding reusability, repeated measurement cycles can lead to the physical entrapment of the redox probe within the polymer matrix or alter the concentration gradients inherent to diffusion-controlled processes. Consequently, regenerating the sensor requires rigorous washing steps that can degrade the MIP layer over time and diminish reproducibility. To overcome reagent dependency while maintaining a faradaic detection mechanism, electroactive species can be integrated directly into the sensor architecture. For instance, redox probes like polymethylene blue (PMB) or ferrocene (Fc) have been successfully immobilized onto carbon-modified electrodes (e.g., MWCNTs) beneath or within the MIP layer [51,78]. In these reagentless setups, techniques like DPV or SWV are used to monitor the intrinsic current of the anchored probe. This current decreases proportionally as target analytes occupy the recognition cavities, physically blocking the localized electron transfer between the immobilized probe and the conductive carbon support.

In addition, completely probeless indirect methodologies have gained significant traction, leveraging techniques such as non-faradaic EIS or capacitive sensing. In these systems, no redox probe is used, and no faradaic electron transfer occurs. Instead, the binding of an electro-inactive analyte into the MIP cavities induces measurable changes in the intrinsic dielectric properties, local capacitance, or electrical conductivity of the polymer layer itself. For example, a conductive eMIP based on polypyrrole (PPy) electrodeposited on laser-induced graphene (LIG) enables highly sensitive, label-free quantification of uncharged analytes like cortisol [99]. Although the analyte lacks charge, its specific binding via hydrogen and hydrophobic interactions alters the ion distribution on the conductive polymer surface, directly modulating the intrinsic conductivity of the PPy/LIG material. This approach allows for direct impedance readouts without any external reagents.

#### 4.2.1. Biomolecules

A representative example of this approach is the detection of bilirubin (BR), the main bile pigment formed during the breakdown of hemoglobin (Hb) in mammals. Bilirubin is an important indicator of liver function and a marker of liver toxicity. Elevated BR levels are associated with severe physiological complications, particularly in newborns, where they can lead to irreversible neurological damage. Therefore, an ultrasensitive eMIP sensor integrated with a ferromagnetic nanocomposite was recently developed for bilirubin analysis in the saliva and serum of newborns. Quantification was indirectly performed by DPV technique using [Fe(CN)_6_]^−3/−4^ as a redox probe. An increase in BR concentration leads to a decrease in peak current due to greater BR adsorption in the imprinted pores on the electrode surface, which reduces the probe’s accessibility to the electrode [113].

The albumin-to-creatinine ratio in urine is another critical clinical parameter for accurately assessing chronic kidney disease (CKD). To evaluate this, an electrochemical sensor was developed based on a dual screen-printed carbon electrode (SPdCE) modified with carboxylated MWCNTs and electropolymerized *o*-phenylenediamine (oPD). This imprinted polymer enables the independent recognition of creatinine and albumin using polymethylene blue (PMB) and ferrocene (Fc) as detection elements, respectively. Measurements performed by SWV in 0.050 M PBS (pH 7.40) showed a linear range from 0 to 50 ng mL^−1^, with LODs of 1.5 ± 0.2 ng mL^−1^ for creatinine and 1.5 ± 0.3 ng mL^−1^ for albumin. The sensor also demonstrated stability for seven weeks at room temperature [51].

Cortisol is another biomolecule that can be indirectly detected on a MIP-based sensor. Its monitoring is of great importance, as chronically elevated levels can trigger anxiety, depression, and cardiovascular diseases. Addressing this analytical need, Ming et al. developed an electrochemical sensor based on an aerogel composed of cellulose nanofibers and MWCNTs. This hybrid material provides a high specific surface area, excellent electrical conductivity, and an appropriate pore size distribution, significantly enhancing the sensitivity of the eMIP. The quantification of the analyte was carried out by DPV, achieving an ultralow LOD of 47.5 fM [75].

Another strategy for the detection of this hormone was developed by Duan et al.*,* based on an eMIP composed of nitrogen-doped carbon nanotubes decorated with nickel nanoclusters. This carbonaceous material served as an anchoring platform for the metal, significantly enhancing its stability. The synthesis of the polymer was carried out using *o*-phenylenediamine (oPD) as functional monomer, where parameters such as the monomer-template ratio, scan rate, and number of polymerization cycles were rigorously optimized in order to control the film thickness and ensure the formation of well-defined cavities. This design exhibited a linear range from 10 fM to 1.0 nM and a LOD of 2.37 fM for DPV measurements, demonstrating superior analytical sensitivity compared to other reported techniques [76].

**Figure 16:**
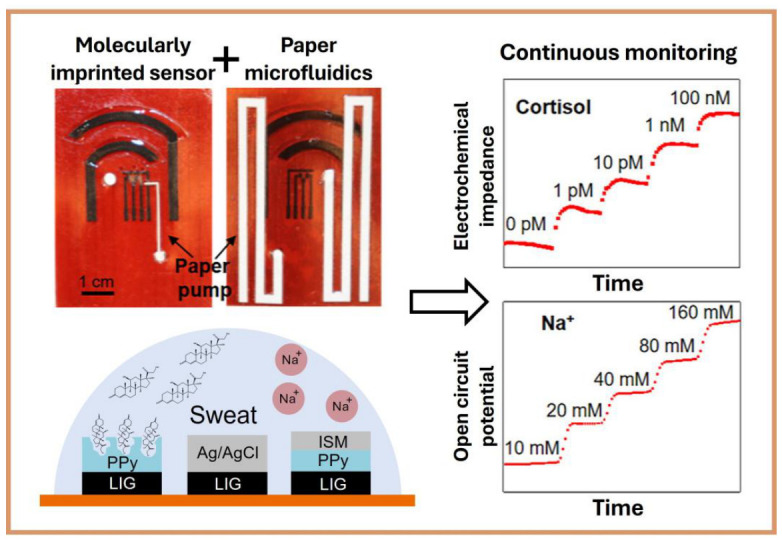
Schematic illustration of the molecularly imprinted wearable sensor design including a cortisol-specific MIP LIG electrode and results of continuous monitoring of cortisol. Reproduced from ref. [99].

In another study, Garg et al. developed a microfluidic wearable device for the continuous and non-invasive monitoring of cortisol in sweat using direct electrochemical impedance spectroscopy (EIS), avoiding the use of redox probes or labels. To achieve this, the eMIP was synthesized on a LIG platform based on polyimide, where the incorporation of a polypyrrole film (100 nm) improved the system’s conductivity by reducing the baseline impedance tenfold compared to the bare LIG. Regarding its analytical performance, cortisol extraction reduced the impedance by half compared to the incubated electrode. Finally, the sensor not only exhibited excellent selectivity against structural analogues but also achieved a LOD of 1 pM in just 3 min, thereby consolidating its viability for clinical applications (Figure 16) [99].

#### 4.2.2. Proteins

Another class of non-electroactive molecules are proteins. Although their large three dimensional structure may complicate some steps of MIP fabrication, their lack of intrinsic electroactivity is resolved also through indirect detection [25]. Among the most widely studied proteins in this field are biomarkers. Specifically, cancer Biomarkers are substances present in blood, body fluid and tissue that provide vital information about the presence, progression or remission of tumors. Carbohydrate Antigen 15-3 (CA 15-3) is a protein produced by normal breast cells as a product of the MUC-1 gene. This carbohydrate is a tumor biomarker for breast cancer, since the level of CA 15-3 in human body increases as this protein is released by tumor cells. To monitor this protein, Oliveira et al. developed a device based on carbon nanotube conductive inks as a support, modified with gold nanoparticles for the subsequent electropolymerization of *o*-phenylenediamine (oPD). This low-cost, disposable screen-printed electrode (SPE) not only enabled mass production and rapid analysis but also highlighted the advantages of MIPs. Unlike immunosensors for this type of biomarker, which, despite possessing higher sensitivity, suffer from poor biological stability, the MIPs demonstrated significantly superior robustness and durability [52].

**Figure 17:**
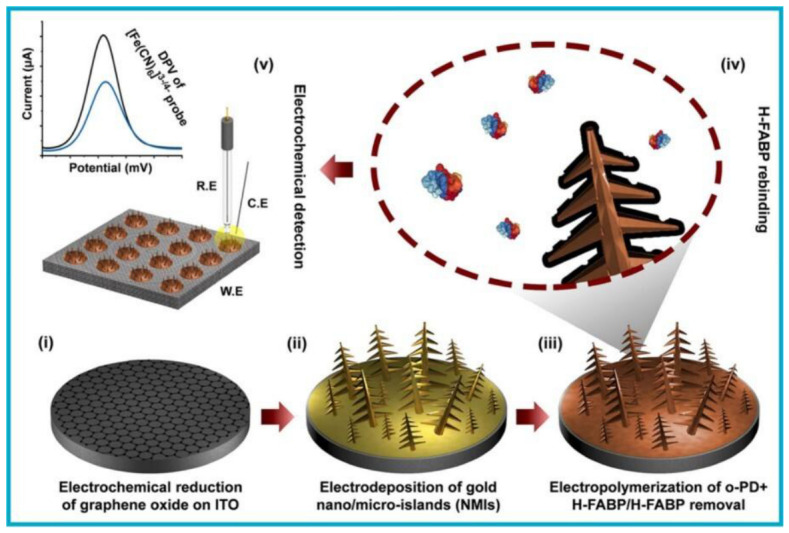
Stepwise schematic of the MIP biosensor fabrication and electrochemical detection of H-FABP. (**i**) Modification of indium–titanium oxide (ITO) surface with GO; (**ii**) electrodeposition of gold NMIs on modified ITO electrode; (**iii**) electrochemical deposition of MIP on NMIs; (**iv**) representation of MIP cavities on the NMI surface; (**v**) changes in electrochemical signal upon H-FABP binding with MIP. Reproduced with permission from ref. [56]. Copyright 2021, American Chemical Society.

On the other hand, Sanati et al. also explored the use of gold nanoparticles to incorporate on carbonaceous surfaces. In this work, they modified FTO electrodes using reduced graphene oxide (rGO) followed by the electrodeposition of gold nanoislands. This device was designed for the detection of heart-type fatty acid-binding protein (H-FABP) (Figure 17). This cardiac biomarker is released into the bloodstream within just 30 to 90 min following an acute myocardial infarction. A notable aspect of this study was the evaluation of the effect of morphology of gold nanostructure on the surface of the sensor, demonstrating that its optimization leads to a significant decrease in the limit of detection. Analytically, the sensor could quantify concentrations below 10 fg/mL in PBS buffer with a response time of 30 s. Furthermore, its clinical applicability was validated by exhibiting satisfactory recovery percentages (between 91.2% and 112.9%) in complex matrices such as human serum, human plasma, and bovine serum, displaying an excellent correlation with the standard ELISA method [56].

Another potential biomarker for cancer is lysozyme (LYS), which is used for the diagnosis of leukemia and various renal or hepatic pathologies. Although its quantification is conventionally performed using classical techniques such as chromatography, electrophoresis, or ELISA, these methods present significant limitations in terms of cost, analysis time, and sample pretreatment. As an alternative, Montoro-Leal et al. developed a sensor based on a magnetic nanocomposite (a magnetite core coated with graphene oxide) as a support for the eMIP. The presence of oxygenated and amino functional groups on the support favored the interaction with the template molecule through hydrogen bonding and dipole interactions during the formation of a microporous polypyrrole film. Using electrochemical impedance spectroscopy (EIS), the change in the charge-transfer resistance (R_ct_) was correlated with the occupation of the imprinted cavities. The sensor achieved a remarkably low LOD of 0.009 pg mL^−1^, a linear range from 1 pg mL^−1^ to 0.1 µg mL^−1^, and an effective reusability of up to 9 cycles [54].

In the field of protein hormones, Pareek et al. addressed the detection of follicle-stimulating hormone (FSH), a key biomarker whose abnormal levels are linked to infertility and polycystic ovary syndrome (PCOS). The authors developed an impedimetric sensor using a MIP supported on a nanocomposite of reduced graphene oxide (rGO) and nickel-cobalt mixed oxide nanoparticles (NiCo_2_O_4_). The incorporation of NiCo_2_O_4_ significantly increased the electrical conductivity and the surface area of the electrode, facilitating the initial electron transfer. The impedimetric sensor for FSH achieved a LOD of 0.1 pM and a sensitivity of 0.57 Ω pM^−1^ [97].

#### 4.2.3. Environmental Pollutants

Although many target analytes are inherently electroactive, their quantification is often performed using indirect detection strategies. This is necessary because certain molecules exhibit high oxidation or reduction overpotentials, requiring the application of extreme potential windows. Operating within these ranges compromises the analytical measurement, as it promotes the simultaneous reaction of interfering species present in the matrix, induces the passivation (or poisoning) of the electrode surface through the adsorption of reaction byproducts, and significantly increases the background noise. In this approach, a well-characterized redox probe is employed to monitor the occupancy of the recognition cavities. This allows the recognition event to be monitored at much more moderate potentials, thereby ensuring the stability and selectivity of the device [64].

Pesticides constitute a critical group of trace contaminants in the food chain. While their extensive use in agriculture is essential to optimize crop yields, their environmental persistence, with residues that can remain active for up to 100 days, poses serious toxicological risks. [118]. In this context, Lakavath et al. developed an impedimetric nanosensor based on a screen-printed electrode modified with reduced graphene oxide for the determination of atrazine, a pesticide whose presence in drinking water is considered toxic above 2 µg mL^−1^ according to the WHO. Because atrazine presents a reduction peak at −0.8 V, a region affected by the oxygen evolution reaction, EIS was employed for its monitoring in presence of 2 mM [Fe(CN)_6_]^3−^/^4−^ redox probe. The continuous occupation of the imprinted cavities blocked the electrode surface, limiting charge transfer. This device achieved a LOD of 100 nM, with a relative standard deviation (RSD) of 3.1% over 26 days [24].

In the quest of portable platforms for the analysis of emerging contaminants, Xu et al. developed a sensor using LIG on a polyimide (PI) substrate. This direct synthesis method generated a three-dimensional structure based on highly porous graphene flakes, providing the electrode with an exceptional surface area and excellent conductivity. The eMIP assembled on this carbonaceous platform successfully quantified perfluorooctanoic acid (PFOA) with an ultralow LOD of 0.025 ppt. Furthermore, the device demonstrated outstanding analytical performance when applied to complex environmental aqueous matrices, including lake, river, and well waters. The combination of its scalability, low manufacturing cost, and compatibility with wireless data transmission systems endows this sensor with enormous potential for continuous and in situ monitoring [114].

#### 4.2.4. Drugs and Pharmaceuticals

In the ongoing pursuit of higher sensitivity and lower limits of detection for the analysis of trace-level contaminants, the use of multiple monomers has gained significant relevance. In this regard, co-electropolymerization has emerged as an effective strategy not only to enhance the structural stability of the MIPs but also to maximize the chemical complementarity of the recognition sites [48,80].

**Figure 18:**
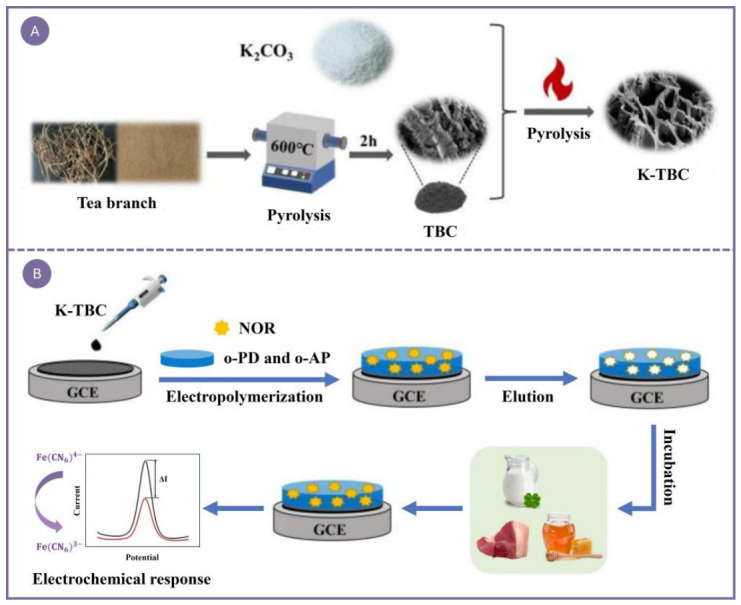
(**A**) Schematic illustration of development of electrochemical sensor for norfloxacin: (**A**) Preparation of tea tree branch biochar and (**B**) Preparation of MIP electrochemical sensor. Adapted from ref. [48].

A group of drugs widely used by both humans and animals are antibiotics, whose excessive consumption can cause toxicity and the development of drug-resistant bacteria. One of these antibiotics is norfloxacin (NOR), which is widely used in livestock, poultry, and veterinary clinics. However, its residues can remain in animal-derived foods and cause serious human health problems. Therefore, Chen et al. developed an eco-friendly electrochemical sensor for NOR detection. Tea branch biochar activated by K_2_CO_3_ was used as a carbonaceous modification on the electrode (Figure 18A), followed by the co-electropolymerization of *o*-PD and *o*-AP (Figure 18B). The biochar provided a high surface area, porosity, good electrical conductivity, and -COOH and -OH functional groups. Meanwhile, the monomers improved the sensitivity, selectivity, and stability of the polymer film due to their high density of functional groups, which promote stronger interactions with the template. The sensor exhibited good analytical performance, achieving a linear range from 0.1 to 100 nM and a stability of 25 days. Furthermore, it obtained satisfactory recovery percentages in milk, pork, and honey samples, ranging from 85.9% to 102% [48].

Sun et al. demonstrated this synergy using *p-*aminobenzoic acid (*p*-ABA) and 2-amino-5-mercapto-1,3,4-thiadiazole (AMT) as functional monomers for the detection of ramipril (RAM), a widely prescribed antihypertensive drug. The authors employed DPV strategy with a [Fe(CN)_6_]^3−^/^4−^ redox probe, as RAM presents a high oxidation potential (+1,37 V), which is severely affected by the water oxidation reaction. They achieved an amplified faradaic current response compared to conventional homopolymers, reaching a LOD up to two orders of magnitude lower. This demonstrated that synergy between multiple monomers significantly enhances the analytical performance of the sensor [69].

Building on the concept of co-polymerization, Zarpour et al. developed an eMIP sensor for tizanidine (TZD) using a nitrogen-doped box-like porous nanostructure substrate to maximize the surface area and optimize charge transfer. On this platform, eMIP was synthesized via the co-electropolymerization of resorcinol (Res) and *o*-aminophenol (*o*-AP). The strategic selection of these monomers lies in their rich hydroxyl and amino groups; their ability to donate and accept protons promotes strong non-covalent interactions (such as hydrogen bonding and π-π interactions) with the template molecule. This synergy generated a uniform, stable, and highly adherent coating with ideal sites for selective recognition. Like the previous study, because TZD exhibits a high oxidation peak (+0.9 V), the authors employed indirect detection to monitor cavity occupancy using DPV in the presence of a redox probe. This device yielded an outstanding LOD of 0.07 nM, outperforming previously reported methods, and achieving recovery percentages close to 100%, demonstrating excellent accuracy and resistance to matrix effects. Furthermore, the sensor exhibited an imprinting factor (IF) of 15.78, evidencing highly specific recognition cavities [115].

### 4.3. Guidelines for Selecting Electrochemical Detection Techniques

The selection of the appropriate electrochemical technique—such as CV, DPV, SWV, linear sweep voltammetry (LSV), and EIS—is not arbitrary; it must be carefully tailored to the sensor architecture and the specific detection mechanism employed. Voltammetric methods, in general, enhance the overall selectivity of the sensor system by identifying the target analyte through its specific oxidation and reduction peaks [65].

Among these, DPV remains the most widely used transduction method [77]. By applying a minor voltage pulse at each potential step, DPV facilitates the differentiation between faradaic and capacitive currents. This effectively minimizes background contributions, yielding an improved signal-to-noise ratio, reduced susceptibility to non-faradaic interference, and exceptional suitability for analysis in complex sample matrices [77,91,100].

For direct detection of electroactive templates, pulse techniques (DPV and SWV) are highly recommended to maximize sensitivity and lower detection limits for trace analysis. Additionally, foundational methods like cyclic voltammetry (CV) and amperometry remain crucial in these direct approaches. Although amperometric sensors based on eMIPs are less common than swept-potential techniques, they offer the distinct advantage of operating at a fixed potential. In these configurations, the continuous reduction or oxidation of the analyte as it binds to the imprinted cavities generates a steady-state current directly proportional to its concentration [65]. This makes amperometry particularly valuable for evaluating the reaction kinetics of diffusion-controlled processes and enabling real-time monitoring.

For indirect detection strategies, the choice depends heavily on the nature of the redox system. When using highly reversible soluble redox probes, such as the [Fe(CN)_6_]^3-/4-^ couple, SWV is particularly advantageous. In SWV, the net current is obtained from the difference between the forward (oxidation) and reverse (reduction) pulses. For highly reversible systems, this creates a synergistic amplification of the faradaic signal, making SWV exceptionally sensitive to minor changes in cavity occupancy. Similarly, for sensors utilizing anchored redox probes (e.g., immobilized PMB or ferrocene) to monitor localized electron transfer, both DPV and SWV provide the necessary resolution.

Furthermore, while EIS is primarily known as a fundamental tool for MIP characterization, it has increasingly emerged as a highly prevalent method for label-free quantitative analysis, especially for non-electroactive analytes [62]. As a powerful and non-invasive electroanalytical technique, EIS provides detailed insights into the recognition event by precisely quantifying microscopic alterations at the electrode surface upon target binding. Specifically, it is highly responsive to capacitive and inductive effects, enabling the precise measurement of changes in charge-transfer resistance (R_CT_) and interfacial capacitance (C_dl_) [62,65,77]. This versatile technique can be applied either by monitoring the R_CT_ of a soluble redox probe or in completely probe-less configurations. In purely resistive (or conductometric) sensors, the specific binding event directly induces a measurable change in the electric resistance—and consequently, the intrinsic conductivity—of the receptor material itself [65]. Because it preserves sample integrity and excels in low-frequency analysis, EIS is uniquely suited for studying complex biological interactions and redox processes involving slow kinetics [77].

### 4.4. Critical Evaluation of Analytical Reporting in eMIP Literature

To conclude this section on (bio)sensing applications, it is crucial to highlight a recurrent analytical shortcoming in the current eMIP literature. While the majority of studies extensively report standard figures of merit, such as limits of detection (LOD), linear ranges, and reproducibility, a significant number of works summarized in Table 2 and Table 3 fail to report the Imprinting Factor (IF) and the Selectivity Factor (α). In the development of molecularly imprinted polymers, the inclusion of a Non-Imprinted Polymer (NIP) is a mandatory control to distinguish specific molecular recognition from non-specific physical adsorption. The omission of the IF in many reports is highly critical, as this parameter is essential to quantitatively validate the successful formation and binding efficiency of the imprinted cavities in the eMIP relative to the NIP. Furthermore, the frequent absence of a rigorously calculated Selectivity Factor limits the ability to confirm true cavity specificity. A robust selectivity evaluation must assess the sensor’s response to various structural analogs and coexisting interferents on both the eMIP and the NIP simultaneously. Without these comparative parameters, assessing the true advantage of the imprinting process becomes challenging. Moving forward, standardizing the reporting of the IF and Selectivity Factor is imperative for the scientific community to accurately benchmark sensor performance and ensure the reliability of eMIP-based devices in complex real-world matrices.

## 5. Conclusions and Future Perspectives

This review provided a comprehensive analysis of electropolymerized molecularly imprinted polymers (eMIPs) supported on carbon-based materials, detailing their entire developmental process from fabrication to application. By exclusively focusing on the electropolymerization route, we highlighted how the precise control over film morphology and thickness, combined with the superior conductivity and high surface area of diverse carbonaceous supports, results in highly sensitive and robust electrochemical sensing platforms.

A central structural pillar of this review is the critical categorization of sensing applications into direct and indirect detection mechanisms. While direct strategies offer simplified, label-free architecture for electroactive targets, indirect approaches (probe-mediated or probe-less) significantly broaden the analytical scope. These indirect methods are essential for detecting non-electroactive species and provide a crucial alternative for electroactive analytes, allowing for highly sensitive detection while avoiding the background interference and material degradation associated with scanning at excessively high anodic potentials.

From a computational standpoint, while DFT has proven invaluable for rationally designing the pre-polymerization complex, its computational cost limits large-scale screening. Looking forward, the integration of machine learning (ML) will revolutionize these initial stages of sensor design. By training ML algorithms on existing thermodynamic and FMO datasets, researchers can significantly accelerate the template-monomer optimization process, instantly predicting the most stable complexes and ideal solvent conditions without the need for time-consuming quantum mechanical calculations. In parallel, the development of “digital twins”—virtual replicas of the electrochemical sensor—will allow researchers to computationally replicate the sensor’s behavior in real time. This approach offers the advantage of addressing inherent operational challenges, such as signal drift, surface fouling, and artifacts, through software rather than physical modifications. Furthermore, by continuously updating with real-time measurements, the system contextualizes raw electrochemical signals, enabling highly accurate, individualized predictive monitoring.

From a materials engineering perspective, future research should explore advanced macromolecular architectures, such as self-healing imprinted polymers, which would significantly extend the sensor’s operational lifespan. Additionally, the shift towards green analytical chemistry demands the exploration of entirely biodegradable carbon supports to mitigate the environmental impact of disposable sensors. The analytical scope must also be broadened through multi-template imprinting strategies, facilitating the multiplexed detection of diverse targets within a single, miniaturized sensing array.

To transition these proof-of-concept devices into real-world tools, seamless integration with modern electronics is paramount. The development of flexible, biocompatible, and implantable eMIP sensors—coupled with miniaturized potentiostates and wireless data transmission systems (e.g., IoT networks and smartphone-based readouts)—will pave the way for continuous, remote, and non-invasive point-of-care monitoring.

Despite these exciting technological horizons, a critical foundational shortcoming remains in the current eMIP literature regarding analytical standardization. As highlighted in our evaluation, there is a frequent omission of fundamental figures of merit, specifically the Imprinting Factor (IF) and rigorously calculated Selectivity Factor (α). Moving forward, it is imperative that the scientific community standardizes the reporting of these metrics by continuously evaluating the eMIP sensor’s performance against a Non-Imprinted Polymer (NIP) control. Coupling this strict analytical rigor with the emerging technologies will be essential for translating laboratory-scale eMIP designs into reliable, commercial, and next-generation devices for modern analytical science.

## Figures and Tables

**Table 1:** Components to the synthesis of MIPs by different methods of polymerization.

Components	Free Radical Polymer	Controlled Radical	Sol–Gel	Electropolymerization
Template	Molecule to imprint	Molecule to imprint	Molecule to imprint	Molecule to imprint
Functional monomer	MAA, acrylamide, acrylic acid	MAA, acrylamide	APTES, MPS	Electroactive monomer (Pyrrole, aniline, oPD)
Crosslinker	EGDMA, TRIM	EGDMA, TRIM	TEOS	Optional
Initiator	AIBN, BPO	AIBN, BPO	Not applicable	No chemical initiator
Activator	Heat or UV light	Heat or UV light (with RAFT agent to control synthesis)	Catalyst and acid or base	Electric potential

MAA: Methacrylic acid; EGDMA: Ethylene glycol methacrylate; TRIM: Trimethylolpropane trimethacrylate; AIBN: 2,2′-Azobis(2-methylpropionitrile); BPO: benzoyl peroxide; APTES: 3-Aminopropyl(diethoxy)methylsilane; MPS: (3-Mercaptopropyl)trimethoxysilane; TEOS: tetraethyl orthosilicate; *o*-PD: *ortho*-phenylene diamine.

**Table 2:** Direct detection.

Carbon-Based Material	Polymerization Conditions	Template	Template Removal	Detection Technique	Performance Parameters	Real Samples	Ref.
**Biomolecules**							
MWCNTs/PGE	Pyrrole CV: −0.6 to +0.9 V, 20 cycles, 100 mV s^−1^	Uric acid	Overoxidation CV: 0.7 to 1.0 V, 30 cycles, 100 mV s^−1^	CV	LR: 0.22 to 3.5 mM LOD: 0.76 mM IF: 6.24	-	[38]
ta-C/CNFs	Pyrrole CV: −0.2 to −0.8 V, 10 cycles, 100 mV s^−1^	Dopamine	Ethanol 50%, 15 min CV: 0.2–0.6 V, 10 mV s^−1^ in PBS 6.0	DPV	LR: 0.01 to 10 μM LOD: 5.4 nM	Horse serum and fetal bovine serum	[70]
Au/N-GOQDs/NiS_2_/kiwi peel-BC/GCE	Nicotinamide CV: −1.0 V to 2.0 V, 25 cycles, 100 mV s^−1^	Dopamine and chlorpromazine (dual sensor)	Methanol/acetic acid (9:1) for 12 min	DPV	Dopamine LR: 0.05 to 8 μM and 8 to 40 μM LOD: 2.8 nM Chlorpromazine LR: 0.005 to 2 μM LOD: 0.25 nM	Human serum, urine and pharmaceutical samples	[102]
Screen printed carbon ink/PET film	Pyrrole CV: 0 V to 1.2 V, 14 cycles, 100 mV s^−1^	Indole-3-acetic acid (IAA)	Overoxidation: 0.9 V for 720 s in NaOH 50 mM and Na_2_HPO_4_ 50 mM	DPV	LOD: 0.1 μM	Tomato fruits and tomato leaves	[106]
**Drugs and pharmaceuticals**							
o-MWCNTs/GCE	Pyrrole 0.75 V vs SCE, 600 s	Sulfamethoxazole	CV: 0.2–1.3 V, 10 cycles, 50 mV s^−1^ BR buffer pH 2.36	DPV	LR: 1.99 μM to 23.4 μM LOD: 413 nM IF: 1.9	Milk	[46]
CS-MWCNTs/GCE	*o*-phenylenediamine CV: −0.1 V to 0.7 V, 20 cycles, 50 mV s^−1^	Chloramphenicol	Methanol/acetic acid (9:1, *v*/*v*) for 15 min	DPV	LR: 0.1 to 1000 μM LOD: 0.033 μM IF: 7.73	Milk	[43]
ZnO-MWCNTs-NH_2_/TCPP/GCE	*o*-phenylenediamine CV: 0 V to 0.8 V, 4 cycles, 50 mV s^−1^	Chlortetracycline	HNO_3_ 2.5% and ultrapure water	Photo- current	LR: 0.5 nM to 10 μM LOD: 0.17 nM	Lake water, milk and pork	[89]
GO/GCE	Aniline CV: −0.2–1.0 V, 7 cycles, 50 mV s^−1^ 2-methoxyaniline CV: −0.2–0.8 V, 7 cycles, 50 mV s^−1^	Amoxicillin	Amperometry: 1.2 V for 300 s in NaOH 0.1 M	SWV	MIP (aniline) LR: 10 to 500 μM LOD: 2.6 μM IF: 3.85 MIP (2-methoxyaniline) LR: 5.0 to 500 μM LOD: 0.61 μM IF: 5.42	Urine and blood plasma	[92]
CNT/MoS_2_-CoNi/GCE	Dopamine CV: −0.2 V to + 0.6 V, 64 cycles, 50 mV s^−1^	Sulfamethazine	Ethanol/acetic acid (1:1) for 20 min	DPV	LR: 0.1 μM to 800 μM LOD: 0.033 μM	Beef and mutton samples	[108]
Au-fMWCNT	Pyrrole CV: 0.0 to +1.3 V, 10 cycles, 50 mV s^−1^	Fluoroquinolone	Overoxidation CV: 0.0 to +1.5 V in PBS (pH 10), and EtOH/NaOH 0.1 M (1:1 *v*/*v*) for 30 min	DPV	LR: 1 to 300 μM LOD: 1 μM	Pharmaceutical tablets and urine sample	[109]
rGO/GCE	3-thiophene acetic acid CV: −0.8 and 0.8 V, 10 cycles, 50 mV s^−1^	Antipyrine and Ethionamide	Methanol/ acetic acid (9:1, *v*/*v*) for 30 min	DPV	Antipyrine LR: 0.05 to 0.6 μM LOD: 0.117 μM LOQ: 0.353 μM Ethionamide LR: 0.03 to 1.2 μM LOD: 0.15 μM LOQ: 0.457 μM	Human blood serum	[63]
Au/rGO/GCE	ZrOCl_2_ CV: −1.2 to 0.0 V, 7 cycles, 20 mV s^−1^	Acetaminophen	Methanol/acetic acid (9:1)	DPV	LR: 0.1 to 100 nM and 0.1 to 4000 μM LOD: 0.1 nM IF: 2.03	Human urine	[91]
CNT/GCE	Pyrrole CV: −0.25 to +0.75 V, 6 cycles, 50 mV s^−1^	Tamoxifen	Overoxidation 1.2 V for 2 min in 0.1 M PBS (pH 6.8)	DPV	LR: 1 to 420 μM LOD: 0.01 μM	Human serum	[60]
MWCNT/GCE	Pyrrole CV: 0.4 V for 30 s and −0.4 to 1 V, 25 cycles, 50 mV s^−1^	Methotrexate	Overoxidation CV: 0.3–1.0 V, 8 cycles	DPV	LR: 0.01 to 25 μM and 25 to 125 μM LOD: 2.7 nM LOQ: 9 nM IF: 3.33	Pharmaceutical formulation and river water	[45]
hNiNS/aMWCNTs @GONRs/GCE	Pyrrole CV: −0.2 V to 0.8 V, 10 cycles, 50 mV s^−1^	Citalopram	Methanol/ acetic acid (9:1) 15 min and CV: 0.2 V to 1.3 V, 10 cycles	DPV	LR: 0.5 to 10 μM and 10 to 190 μM LOD: 0.042 μM	Tablet, human urine and blood serum	[81]
ZMO/MWCNT/GCE	Dopamine CV: −0.4 to +1.6 V, 15 cycles, 100 mV s^−1^	Chlorpromazine	CV: −0.4 to 1.6 V and −0.2 to 1.2 V	DPV	LR: 0.002 to 0.1 μM and 0.3 to 7.5 μM LOD: 0.42 nM IF: 1.58 and 7.08	Serum, urine and lake water	[86]
NSC/CC	Cu-coordinated pyrrole-3-carboxylic acid (Cu-PyCOOH) CV: −0.2 V to 1.2 V, 15 cycles, 100 mV s^−1^	Perphenazine	Methanol for 30 min	DPV	LR: 1 to 700 nM LOD: 0.43 nM IF: 2.67	Tablets and human serum	[104]
CNTs/GCE	Aniline CV: +0.15 to +1.1 V, 5 cycles, 20 mV s^−1^	Stanozolol	Methanol/ acetic acid (9:1) for 5 min	DPV Amperometry	LR: 0 to 120 µM LOD: 0.009 ng mL^−1^	Blood plasma	[37]
AuNPs/PGE	Dopamine CV: −0.2 V to +0.8 V, 15 cycles, 50 mV s^−1^	Empagliflozin	Methanol/acetic acid (1:1)	DPV	LR: 0.005 to 1.00 μM and 1.30 to 100 μM LOD: 1.19 nM LOQ: 3.60 nM	Tablets, human plasma, and urine	[107]
**Environmental pollutants**							
SWCNT/GCE	*o*-aminophenol CV: −0.5 to 1.5 V, 6 cycles, 50 mV s^−1^	Zineb fungicide	Nitric acid/ ethanol (1:2) for 6 min	DPV	LR: 5 to 1000 nM LOD: 1.6 fM IF: 40.30	Vegetables and fruits	[42]
Co/M_2_C/N-CNT	*o*-phenylenediamine CV: −0.2 to +0.80 V, 10 cycles, 80 mV s^−1^	Imidacloprid	Methanol/acetic acid (9:1) for 16 min	DPV	LR: 0.1 to 100 μM LOD: 0.033 μM	Tea	[110]
CNTs/GCE	Melamine CV: 0–1.0 V, 30 cycles, 50 mV s^−1^	Hydroquinone and catechol	Ethanol/water (1:1, *v*/*v*) for 5 min	DPV	Hydroquinone LR: 10 to 100 μM LOD: 3.1 μM Catechol LR: 10 to 100 μM LOD: 3.5 μM	River water	[90]
MnCO_3_NS/CF/GCE	Pyrrole CV: 0.00 to +1.00 V, 20 cycles, 100 mV s^−1^	Ochratoxin A	NaCl 1.0 M	DPV	LR: 0.01 to 1.0 nM LOD: 2.0 pM LOQ: 10.0 pM	Apple juice	[53]
Pt/PgCN/CFP	3-thiopheneacetic acid CV: −0.4 V–1.6 V, 20 cycles, 50 mV s^−1^	Butylated hydroxy anisole	Overoxidation: 500 s in PBS 0.2 M	DPV	LR: 0.5 to 210 nM LOD: 5.83 nM.	Flour	[105]
**Industrial dyes**							
MWCNT/GCE	Pyrrole CV: −0.20 to 0.80 V, 5 cycles, 0.1 V s^−1^	Amaranth	Overoxidation: 1.3 V for 120 s in 0.1 M PBS (pH 6.0)	LSV	LR: 0.007 to 1.00 μM and 0.4 to 17.0 μM LOD: 0.4 nM	Fruit drinks	[61]
ZnO/CPE	Arginine CV: −2.2 to 2.0 V, 12 cycles, 100 mV s^−1^	Tartrazine	Ethanol and water (1:1) for 15 min	DPV	LR: 0.008 to 0.112 μM and 0.25 to 5.0 μM LOD: 0.0027 μM IF: 8	Soft drink, orange flavored and jelly powder	[111]

ZMO: ZnMn_2_O_4_; ta-C: tetrahedral amorphous carbon; N-GOQDs: Nitrogen-doped graphene oxide quantum dots; BC: biomass carbon; hNiNS/aMWCNTs@GONRs: Hollow nickel nano-spheres/activated multiwalled carbon nanotubes@graphene oxide nanoribbons; CC/NSC: Flexible porous carbon cloth with nitrogen and sulfur co-doped porous carbon; MnCO_3_NS/CF: MnCO_3_ nanostructures incorporated into carbon fibers; Pt/PgCN/CFP: Platinum-decorated phosphorous doped graphitic carbon nitride in carbon fiber paper.

**Table 3:** Indirect detection.

Carbon-Based Material	Polymerization Conditions	Template	Template Removal	Detection Technique	Performance Parameters	Real Samples	Ref.
**Biomolecules**							
AuFe_2_O_3_-GrCNT/GCE	*o*-aminothiophenol CV: −0.2 to 0.6 V, 10 cycles, 50 mV s^−1^	Bilirubin	Ethanol and HCl 0.5 M for 10 min	DPV [Fe(CN)_6_]^−3/−4^ solution	LR: 3.7 to 13.2 nM and 3.7 to 13.0 pM LOD: 1.54 nM and 1.36 pM LOQ: 5.14 nM and 5.3 pM	Saliva and blood serum	**[113]**
Fc/c-MWCNTs/SPdCE and PMB/c-WCNTs/SPdCE	*o*-phenylenediamine CV: 0.00 to +0.80 V, 50 mV s^−1^, 10 cycles (Albumin) and 3 cycles (Creatinine)	Albumin and Creatinine	Oxalic acid 0.10 M for 60 min	SWV PBS (pH 7.40) solution	Creatinine LR: 5.0 to 100 μM and 0.1 to 2.5 mM LOD: 1.5 ± 0.2 μM LOQ: 5.1 ± 0.7 μM Albumin: LR: 5.0 to 100 μM LOD: 1.5 ± 0.3 μM LOQ: 5.2 ± 0.3 μM	Human urine	[51]
NiNCs-N-CNTs/GCE	*o*-phenylenediamine CV: 0.0 to 0.8 V, 20 cycles, 75 mV s^−1^	Cortisol	NaOH 0.1 M CV: −1.0 V to +1.0 V, 3 cycles	DPV [Fe(CN)_6_]^−3/−4^ solution	LR: 10 fM to 1 nM LOD: 2.37 fM	Saliva	**[76]**
CCA/GCE	*o*-phenylenediamine CV: 0 to 1 V, 30 cycles, 50 mV s^−1^	Cortisol	Ethanol (95%) for 15 min	DPV [Fe(CN)_6_]^3−/4−^ solution	LR: 50 fM to 10 μM LOD: 47.5 fM	Saliva and artificial sweat	**[75]**
LIG	Pyrrole CV: −0.2 to 0.9 V, 10 cycles, 50 mv s^−1^	Cortisol	Acetic acid/methanol (7:3) for 30 min	EIS [Fe(CN)_6_]^3−/4−^ solution	LR: 0.1 pM to 1 μM LOD: 1 pM	Sweat	[99]
PGE/GO	2-(dimethylamino) ethyl methacrylate CV: −1.0 to +1.0 V, 20 cycles, 100 mV s^−1^	Cholesterol	Overoxidation CV: −1.0 to +1.0 V, 20 cycles, 100 mV s^−1^	DPV [Fe(CN)_6_]^3−/4−^ solution	LR: 1 to 6 mM LOD: 0.85 mM LOQ: 2.85 mM IF: 3.36	-	**[93]**
MnxOy NPs/rGO/GCE	L-Serine (L-Ser) CV: −0.3 V a 1.3 V, 10 cycles, 50 mV s^−1^	Palmitic acid	Overoxidation CV: 0 V to 1.5 V, 6 cycles, 50 mV s^−1^ in NaOH 0.1 M	DPV [Fe(CN)_6_]^3−/4−^ Solution	LR: 2.0 to 10 pM and 10 to 100 pM LOD: 0.86 pM and 2.0 pM IF: 2.12	Guava seed oil	**[96]**
LIG	3,4-ethylenedioxythiophene (EDOT) CV: −0.7 V to +1.1 V, 10 cycles, 100 mV s^−1^	Lactate	Acetic acid/methanol (3:7) for 10 min	SWV [Fe(CN)_6_]^3−/4−^ Solution	LR: 0.1 to 1000 μM LOD: 0.033 μM	Artificial saliva	**[100]**
**Proteins**							
MWCNTs/GCE	Phenol CV: −0.2 to 1 V, 20 cycles, 70 mV s^−1^	Growth hormone-releasing hexapeptide (GHRP-6)	CV: −1 V to 1 V, 15 cycles, 50 mV/s in NaOH 0.1 M	DPV [Fe(CN)_6_]^3−/4−^ Solution	LR: 0.1 to 100 ng mL^−1^ LOD: 0.0012 ng mL^−1^ IF: 19.08	Human serum	**[41]**
CNT ink/AuNPs	*o*-phenylenediamine CV: −0.2 to 1.0 V, 20 cycles, 50 mV s^−1^	Cancer Biomarker CA 15-3	Oxalic acid 10 mM in PBS 0.1 M (pH 7.5)	Chrono- amperometry K_4_[Fe(CN)_6_] solution	LR: 5 to 35 U mL^−1^ LOD: 1.16 U mL^−1^ LOQ: 3.87 U mL^−1^	Serum and saliva	**[52]**
GO@Fe_3_O_4_/GE	Pyrrole CV: −0.6 V to 1.2 V, 1 cycle, 100 mV s^−1^	Lysozyme	H_2_SO_4_ 0.5 M for 1 h	EIS [Fe(CN)_6_]^3−/4−^ Solution	LR: 1 to 1 × 10^5^ pg mL^−1^ LOD: 0.009 pg mL^−1^ LOQ: 0.9 pg mL^−1^	Chicken egg white and commercial drug	**[54]**
AuNMIs/rGO/ITO	*o*-phenylenediamine CV: 0 to 0,8 V, 20 cycles, 50 mV s^−1^	Heart-fatty acid binding protein (H-FABP)	Ethanol/water (5:1) NaOH 0.1 M	DPV [Fe(CN)_6_]^3−/4−^ Solution	LR: 1 fg mL^–1^ to 100 ng mL^–1^ LOD: 2.29 fg mL^–1^	Human serum and plasma, and bovine serum	**[56]**
PMB/MWCNTs/SPCE	Aniline CV: −0.20 and +1.0 V, 10 cycles, 50 mV s^−1^	Cardiac troponin T (cTnT)	Acetic acid 0.5 M for 4h	DPV Polymethylene Blue Immobilized	LR: 0.10 to 8.0 pg mL^−1^ LOD: 0.040 pg mL^−1^	Blood plasma	**[78]**
NiCo_2_O_4_/rGO/ITO	Methyl methacrylate CV: −0.2 to +0.8 V, 20 cycles, 50 mV s^−1^	Follicle-Stimulating Hormone (FSH)	Ethanol/H_2_O (1:1) for 6 min	EIS [Fe(CN)_6_]^3−/4−^ Solution	LR: 0.1 pM to 1 μM LOD: 0.1 pM	Human blood	**[97]**
NCD-G/SPCE	Pyrrole CV: 0 to +0.8 V, 3 cycles, 100 mV s^−1^	Amyloid-β42 protein (Aβ42)	Oxalic acid 5 mM for 10 min	SWV [Fe(CN)_6_]^3−/4−^ Solution	LR: 5 to 70 pg mL^−1^ LOD: 1 pg mL^−1^	Artificial human serum	**[103]**
**Environmental pollutants**							
g-C_3_N_4_/GCE	Dopamine CV: −0.5 V to +0.5 V, 10 cycles, 20 mV s^−1^	Asulam	Etanol/HNO_3_/H_2_O (2:3:5) for 6 min	DPV [Fe(CN)_6_]^3−/4−^ Solution	LR: 0.5 to 20 pM LOD: 0.17 pM IF: 15	-	**[40]**
NiCo_2_O_4_/MWCNTs/GCE	*o*-phenylenediamine CV: −0.1 to 0.9 V, 30 cycles, 50 mV s^−1^	Dimethoate	Methanol	DPV [Fe(CN)_6_]^3−/4−^ Solution	LR: 0.05 to 10,000 nM LOD: 18.52 pM IF: 5.564	Fresh oranges and cucumbers	**[84]**
GO/SPE	Methacrylic acid (MAA) CV: −0.2 V to 0.6 V, 15 cycles, 50 mV s^−1^	Benzene	Methanol/acetic acid (4:1)	CV [Fe(CN)_6_]^3−/4−^ Solution	LR: 0.1 to 1000 ppb S: 15.5 μA/ppb	Liquid benzene	**[95]**
rGO/SPCE	Pyrrole CV: −1.5 to 1.2 V, 10 cycles, 50 mV s^−1^	Atrazine	Ethanol/H_2_SO_4_ for 60 min	EIS [Fe(CN)_6_]^3−^ Solution	LR: 0.5 to 10 μM LOD: 0.1 μM	Tap water, tomato, capsicum, cabbage	**[24]**
LIG	*o*-phenylenediamine CV: 0 to 0.8 V, 20 cycles, 50 mV s^−1^	Perfluorooctanoic acid (PFOA)	Methanol/water (1:1) for 20 min	DPV [Fe(CN)_6_]^3−/4−^ Solution	LR: 0.025 ppt to 25 ppb LOD: 0.025 ppt	Lake, river, and well water	**[114]**
**Drugs and pharmaceuticals**							
K-TBC/GCE (tea branch biochar)	*o*-phenylenediamine and o-aminophenol CV: 0 to 1.2 V, 30 cycles	Norfloxacin	NaOH/Ethanol (1:3) for 12 min	DPV [Fe(CN)_6_]^3−/4−^ Solution	LR: 0.1 to 0.5 nM and 0.5 to 100 nM LOD: 0.028 nM	Milk, honey and pork	**[48]**
SnS_2_-MWCNTs/GCE	p-aminobenzoic acid and 2-amino-5-mercapto-1,3,4-thiadiazole CV: −0.2 V to +1.7 V, 16 cycles, 50 mV s^−1^	Ramipril	Ethanol/acetic acid (1:1) for 12 min	DPV [Fe(CN)_6_]^3−/4−^ Solution	LOD: 23.9 fM LOQ: 79.7 fM	Tritace tablets	**[69]**
c-MWCNTs-ZIF/GCE	Nicotinamide and 2-amino-5-mercapto-1,3,4-thiadiazole CV: −0.6 to 1.8 V, 40 cycles, 125 mV s^–1^	Nitrofurazone	Methanol/acetic acid (9:1) for 15 min	DPV [Fe(CN)_6_]^3−/4−^ Solution	LR: 0.1 pM to 1 μM LOD: 67 fM	Urine and water	**[80]**
WS_2_/MWCNTs-COOH/SPE	*o*-phenylenediamine CV: 0.0 to 1.0 V, 30 cycles, 50.0 mV s^−1^	Chlortetracycline	Ethanol for 15 min	DPV [Fe(CN)_6_]^3−/4−^ Solution	LR: 0.5 to 425.0 μM LOD: 18.5 nM	Tap, river and sea water, pharmaceutical wastewater, and domestic sewage	**[82]**
N-C n-Boxes/GCE	Resorcinol and 2-aminophenol CV: −1.0 to 1.0 V, 14 cycles, 20 mV s^−1^	Tizanidine	Methanol/acetic acid (3:5) for 140 s	DPV [Fe(CN)_6_]^3−/4−^ Solution	LR: 0.1 ng mL^−1^ to 10 μg mL^−1^ LOD: 0.073 ng mL^−1^ IF: 15.7	Blood serum and urine samples	**[115]**
Peanut shell-AC/CPE	*o*-phenylenediamine CV: 0 to 1,0 V, 10 cycles, 100 mV s^−1^	Letrozole	Methanol/HCl 1.0 M (3:2) for 3 min	DPV [Fe(CN)_6_]^3−/4−^ Solution	LR: 1.0 to 38.0 pM LOD: 0.32 pM LOQ: 0.98 pM IF: 15.59	Human plasma	**[116]**
**Food security**							
MWCNTs/CPE	Pyrrole CV: −0.5 to 1.5 V, 20 cycles, 100 mV s^−1^	Sodium dehydroacetate	Methanol/acetic acid (9:1) for 4 min	DPV [Fe(CN)_6_]^3−/4−^ Solution	LR: 4.1 nM to 1.2 μM LOD: 0.13 nM LOQ: 0.43 nM IF: 16.6	Strawberry juice	**[85]**
AuNPs/rGNRs/GCE	*o*-phenylenediamine CV: −0.2 to 1.3 V, 30 circles, 100 mV s^−1^	Zearalenone	Methanol/acetic acid/ultrapure water (7:3:5) for 45 min	DPV [Fe(CN)_6_]^3−/4−^ Solution	LR: 1 to 500 ng mL^−1^ LOD: 0.34 ng mL^−1^	Maize flour	**[98]**
rGO/GCE	Aspartic acid CV: −1.5 V to 2.0 V, 3 cycles, 100 mV s^−1^	Sucrose (SUC)	Acetonitrile/acetic acid (9:1) for 20 min	DPV [Fe(CN)_6_]^3−/4−^ Solution	LOD: 2.7 fM LOQ: 8.1 fM IF: 3.43	Sugarcane juice	**[117]**

Fc: Ferrocene; NiNCs-N-CNTs: Nickel nanoclusters-loaded nitrogen-doped carbon nanotubes; CCA: Cellulose nanofiber multiwall-carbon nanotube composite aerogel; AuNMIs: gold nano/micro-islands; PMB: Poly-methylene blue; NCD-G: Nitrogen-doped carbon-dot-graphene nanohybrid; g-C_3_N_4_: graphitic carbon nitride; ZIF: zeolitic imidazolate frameworks; K-TBC: Potassium carbonate activated tea branch biochar; N-C n-Boxes: Nitrogen-doped carbon Nanoboxes; rGNRs: reduced graphene nanoribbons.

## Data Availability

No new data were created or analyzed in this study. Data sharing is not applicable to this article.

## Abbreviations

The following abbreviations are used in this manuscript:

MIP	Molecular Imprinted Polymer
GO	Graphene Oxide
rGO	Reduced Graphene Oxide
CNT	Carbon Nanotubes
SWCNT	Single-Walled Carbon Nanotubes
MWCNT	Multi-Walled Carbon Nanotubes
c-MWCNT	Carboxilated Multi-Walled Carbon Nanotubes
o-MWCNT	Oxidized Multi-Walled Carbon Nanotubes
SPCE	Screen-printed Carbon Electrode
SPdCE	Screen-printed dual Carbon Electrode
CQDs	Carbon Quantum Dots
AFM	Atomic Force Microscopy
SEM	Scanning Electronic Microscopy
FTIR	Fourier Transform Infrared
CV	Cyclic Voltammetry
DPV	Differential Pulse Voltammetry
SWV	Square Wave Voltammetry
EIS	Electrochemical Impedance Spectroscopy
